# Intolerance of Uncertainty as a Central Influence on Social Media Use: A School-Based Program for Adolescents

**DOI:** 10.1007/s11121-024-01738-y

**Published:** 2024-10-25

**Authors:** Danielle A. Einstein, Anne McMaugh, Ronald M. Rapee, Peter McEvoy, Madeleine I. Fraser, Maree Abbott, Warren Mansell, Eyal Karin

**Affiliations:** 1 School of Psychological Sciences, Macquarie University, Sydney, NSW Australia; 2 Macquarie School of Education, Macquarie University, Sydney, Australia; 3 Faculty of Human Sciences, Macquarie University, Macquarie University, Sydney, Australia; 4 Curtin University, Perth, Australia; 5 Australian Catholic University, Sydney, Australia; 6 Faculty of Science, The University of Sydney, Sydney, Australia

**Keywords:** Intolerance of Uncertainty, Adolescents, Cognitive Behaviour Therapy, ACT, Universal prevention, Transdiagnostic

## Abstract

This study evaluated a classroom program to address Intolerance of uncertainty (IU) in secondary school students in Australia. IU is a transdiagnostic vulnerability factor for a range of psychological difficulties. A universal classroom program aimed to increase student understanding of feelings of uncertainty and explored socioemotional factors that could influence behaviors when facing uncertainty. Classroom teachers in two high schools were trained to deliver an 18-lesson program under naturalistic classroom conditions. Relative to the control group, the intervention lowered IU at post-test, but not at a later follow-up. The only socioemotional outcome measure to demonstrate a concurrent change was social media use. Limitations of the study were associated with the naturalistic classroom intervention, including the high level of attrition. Future studies may need to address broader factors in the students’ context that could influence intolerance of uncertainty.

**Supplementary Information:**

The online version contains supplementary material available at 10.1007/s11121-024-01738-y.

Intolerance of Uncertainty (IU) is a transdiagnostic risk factor for a range of emotional disorders including neuroticism (Carleton, 2016), worry (Thielsch et al., 2015), and negative affect (Janssen et al., 2020). At lower levels, IU is associated with hardiness, a personality trait that reflects one’s ability to manage stressful situations and supports the individual to maintain internal balance without compromising performance (Andronnikova, 2021).

In an extension of the transdiagnostic model of IU, Einstein (2014) posited that Prospective IU was a “gate keeper” for emotional symptoms with low levels of Prospective IU being associated with less psychopathology. Based on this theoretical proposition, lowering an individual’s “Need for Predictability” (Prospective IU) should lead to improvements and less maladaptive responses (e.g., safety behaviors, reassurance seeking, social media use) when facing uncertainty. Prospective IU reflects the meta-belief that the individual needs to know what will happen in the future, which is impossible and therefore creates conflict. Both the desire for predictability and its’ consequent creation of conflict render the individual less flexible and more dependent in response to stressors. For example, when presented with an uncertain situation, an individual may seek reassurance to reduce the potential threat (Rapee, 1985). However, reassurance may only temporarily relieve distress as complete certainty cannot be guaranteed. Newman and Llera (2011) proposed that individuals reporting Prospective IU were less flexible psychologically, preferring to avoid the emotional shift brought about by unpredictable events and seeking certainty even though it may not be attainable prior to an event. 

Experimental results suggest the pivotal role of Prospective IU in emotional regulation. When uncertainty was induced, individuals with high Prospective IU were unable to distract from a topic of concern and described themselves “becom(ing) a control freak,” demonstrating overutilization of plans and fall-back strategies (Bottesi et al., 2019, p.64). In a second study, Ranney et al. (2019) found that individuals with high trait Prospective IU believed that gathering information about uncertain events (i.e., an upsetting film clip) would reduce their distress; however, the information did not reduce their distress.

In both adolescents and adults, IU reductions are associated with treatment response to interventions (McEvoy & Erceg-Hurn, 2016; Palitz et al., 2019; Rifkin, 2022; van der Heiden et al., 2012)^1^. Targeting IU during the treatment of excessive worry for adolescents can be effective, particularly when parents are involved (Palitz et al., 2019; Wahlund et al., 2020; Yildiz & Iskender, 2021). When parents are not involved, for example in a universal classroom program, there remains a question about whether it is possible to lower IU. If IU can be reduced in a large group within a school setting, the second important question is whether lowering IU in adolescents would prevent maladaptive coping and positively affect their wellbeing.

There are potential risks and limitations of exploring psychological factors within a classroom prevention program. First, classroom programs might involve personal disclosures at a time when peer relationships can be turbulent and bullying increases (Bellmore et al., 2017; Meter & Card, 2016). Second, while teachers can deliver socioemotional programs in the classroom, implementation problems can limit intervention effectiveness (Durlak et al., 2011). Third, there are individual differences in mental health and in responses to content delivered within any classroom. Therefore, a universal prevention program may not benefit all students and emotional problems may be exacerbated, as occurred in the large-scale mindfulness universal prevention effectiveness study (Kuyken et al., 2022; Montero-Marin et al., 2022). Thus, a universal prevention program requires attention to outcomes at the universal level as well as attention to the outcomes of vulnerable students.

The primary aim of the study was to evaluate the efficacy of a universal classroom intervention to lower Prospective and Inhibitory IU over the course of the program and at follow-up. A related aim was to examine changes in social media use over the course of the program. Problematic social media use and IU are linked with studies demonstrating direct associations and mediated effects on other mental health factors such as anxiety (Reed & Haas, 2023; Sun et al., 2022). We would hope to see a reduction in problematic social media use in adolescence as an important behavioral outcome of this intervention. The final aim of this study was to explore the efficacy of the program for the most vulnerable students who provided baseline reports indicative of the top 20th percentile of concern in any given measure.

## Method

### Design

This quasi-controlled trial was registered with ANZCTR with a naturalistic design reflecting the pragmatic constraints of operating within a school environment (Table 1). The two participating schools in this report provided an intervention group and a control group. They each delivered the intervention within a single calendar year. Schools selected the year group that would receive the prevention program. Schools nominated the intervention dosage rate that could be accommodated within their calendar which varied between 6 months and one calendar year. Only data from students whose parents had provided consent forms were included in the data analysis. The control condition received the standard physical development and health education curriculum.

**Table 1:** Implementation differences across schools

School	Condition (*N*)	Supervision and dosage	Post (months)*	Follow-up (months)*
1	Control (70)	School counsellor; Weekly, 1 year	2.53 (0.01)	13.53 (0.04)
1	Insights (383)		9.99 (0.13)	22.32 (0.09)
2	Control (76)	CI (2016)^**^ Twice/week, 6 months	3.34 (0.1)	12.79 (0.11)
2	Insights (74)		6.57 (0.09)	15.97 (0.17)

*Months elapsed since baseline surveys were administered. **CI Chief Investigator 2016 teacher supervision

### Participants

Participants were Year 8 and 9 students attending two schools. Both schools were private schools with higher than average scores on the Index of Community Socio-Educational Advantage (ICSEA). The ICSEA is a composite sociodemographic score, indicative of the educational advantage of the school population, calculated and reported for each school by the national curriculum and reporting authority (ACARA, 2015). School 1 was a coeducational school located in a large regional city (see Table 1). School 2 comprised only girls and was located in a large city.

### The Insights Prevention Program

**Fig. 1:**
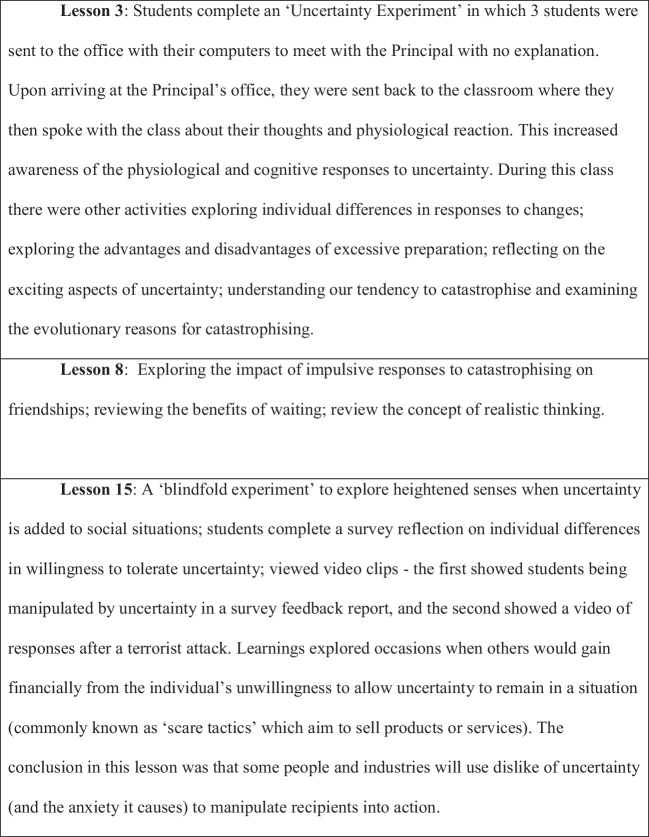
Intervention protocol for uncertainty psychoeducation lessons

The Insights program (Einstein et al., 2016) used the theory and principles of psychoeducation, Acceptance and Commitment Therapy, and Cognitive Behavior Therapy to target a range of social and emotional factors relevant to how secondary school students respond to uncertainty. The program was comprised of 18 lessons which aimed to change beliefs around uncertainty. Three lessons specifically addressed feelings of uncertainty with psychoeducation (Fig. 1), while the remaining 15 lessons reinforced these concepts within lessons which related these ideas to relevant behaviors and thoughts (e.g., responding to uncertainty with urgency via text messages when feeling upset, catastrophizing, facing fears, respectful relationships).

Overall, the 18-lesson Insights program addressed concepts believed to be topical and crucial to social and emotional development in this age cohort. Specific skills taught included self-soothing, problem solving, and accepting emotions.

Figure 1 displays the intervention protocol for the three uncertainty psychoeducation lessons. Treatment fidelity and dosage rates were maintained for 28% of students during delivery whereby staff indicated which components were included in each lesson and which students attended each lesson.

### Measures

The primary outcome of Intolerance of Uncertainty was targeted in the program while multiple secondary outcomes were also assessed and are presented in the supplementary results.

#### Intolerance of Uncertainty

The Intolerance of Uncertainty Scale (IUS-12; Short form; Carleton et al., 2007) is a 12-item scale measuring two dimensions of Prospective and Inhibitory intolerance of uncertainty (IU), rated on a 5-point Likert scale. The Prospective IU subscale has seven items (*α* = 0.73) assessing the level to which the individual wishes to avoid surprises and their desire to be organized and in control of future happenings (e.g., One should look ahead so as to avoid surprises). The Inhibitory IU subscale has five items (*α* = 0.81) and measures the amount of paralysis induced by uncertainty for the individual (e.g., “When I am uncertain I can’t function very well”; Boelen et al., 2010; Carleton et al., 2007).

#### Secondary Outcome Measures

##### Social Media Items

Social media items were drawn from a range of separate scales. The first set of items were validated in a large Australian adult and adolescent sample using the Australian Psychological Society Stress and Wellbeing Survey (Australian Psychological Society, 2015). These items assess whether participants experience discomfort and Fear of Missing Out (FoMO) when disconnected from social media. A second set of items examined the style of social media use for example “On social media how often do you: write a status update, post your photos, read the newsfeed…” (Tandoc et al., 2015). FoMO was assessed using items from Przybylski et al. (2013) including “I am afraid that I will miss out on something if I don’t stay connected to my online social networks. I feel worried and uncomfortable when I can’t access my social media accounts.” Self-report measures have been widely used in previous studies assessing the frequency of social media use (e.g., Pantic et al., 2012). The trial commenced using a 5-point Likert scale from 1 (Never) to 5 (Twice daily or more). In the second year of the trial, device use had become more widely adopted, and the research team felt that the response scale should be substituted for a 1 to 8 scale where 1 remained “Never” and 8 became “Constantly.” In the treatment of the full data set, social media responses from the 5-point Likert scale were re-standardized using the mean and standard deviation of the 8-point Likert scale. In order to understand the impact of the program on particular facets of social media use, several subscales were developed from these items. These are detailed in Supplementary Table 9.

The remaining secondary outcome measures were established self-report measures with demonstrated reliability and validity in adolescent populations. These are listed in Supplementary Table 2. They were included to examine the effects of the program on a range of vulnerabilities.

### Procedure

The study received ethical approval from the Macquarie University Human Ethics Committee. Participation required the return of a signed consent form from both the student and a parent prior to data collection. In total, 692 (70%) students provided consent to participate in the study, and 603 (61%) students were present at school to complete the baseline surveys. These participants comprised the final study sample (*n* = 603).

### Statistical Analyses

The intervention was evaluated in three ways. Firstly, analyses of change over time examined condition-related differences (prevention vs. control) in the rate of outcome change over the time windows of pre-treatment to post-treatment (16–40 weeks from pre-treatment), and through to the follow-up period (52–104 weeks from pre-treatment). To test the rate of change between conditions, a series of Generalized Estimation Equation (GEE) models were used to examine the average group change in all the primary and secondary outcomes. Each model was specified with an unstructured working correlation and robust error estimation to account for the within-subject variance of repeated measurement over time (Hubbard et al., 2010; Liang & Zeger, 1986). To examine the change in outcomes, we utilized estimated marginal means, percentage change estimates, and Hedges’ g metric to assess within-condition change over time. We applied the reliable change index (Jacobson & Truax, 1991) to categorize intra-individual changes into deterioration (an increase of 11 points from baseline), non-response (a change within an 11-point reduction and an 11-point increase from baseline), and remission (a reduction of more than 11 points from baseline). This categorization was used to convey the rates of possible either adverse or beneficial events as proportions of the sample based on 95% confidence intervals.

In a second step, a series of sensitivity analyses was conducted to examine if the rate of outcome change between the groups varied based on student characteristics such as age, gender, and presentation of increased symptoms at baseline (e.g., children in the top 20th percentile or higher for each outcome). In addition, treatment fidelity, indicated by dosage rate or amount of module completion (less than half; half or more; complete) attendance at IU lessons, was considered. These analyses aimed to assess the sensitivity of the intervention (time-by-group effects) for generalizability across different ages and genders, to evaluate the specificity of treatment effects for children with initially elevated symptoms (relevance effects), and to examine the impact of varying intervention dosages (dosage specificity effects).

Analyses were conducted in SPSS version 28 (SPSS, Chicago, IL) and assumed the adjusted type I error rate of *p* < 0.01 for interpreting test significance. This was set to be conservative but not overly restrictive. This error rate was more conservative than universal prevention studies which have used *p* < 0.05 (Dray et al., 2017). Additionally, in line with intention-to-treat principles (Hollis & Campbell, 1999), models included adjustments for any data lost over time. Missing data were addressed using a conditional multiple imputation procedure under the assumption of conditional Missing at Random (MAR).

## Results

### Sample Attained

**Fig. 2:**
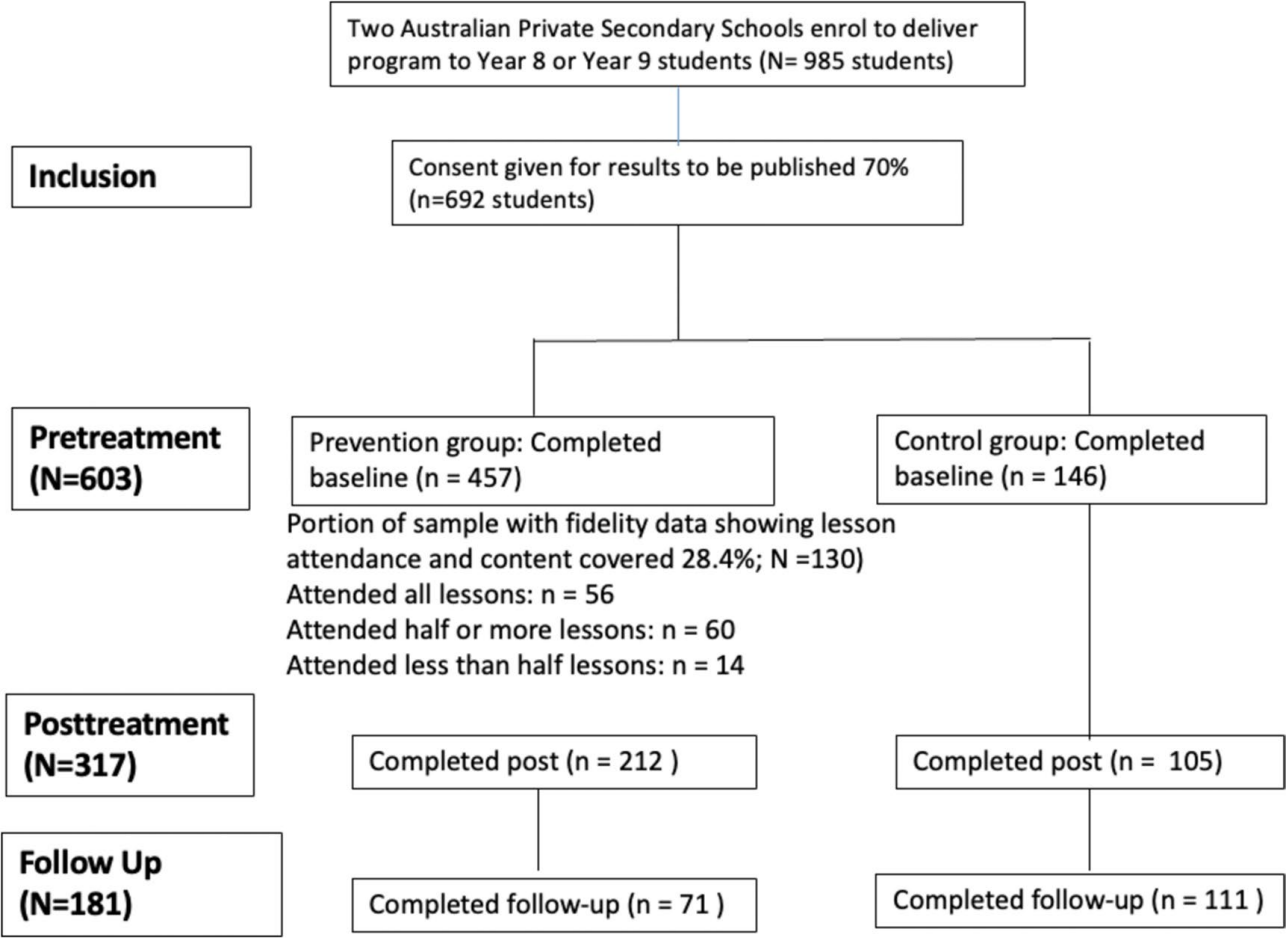
Study design

Table 2 and Supplementary Table 1 display the baseline demographic and clinical characteristics of the sample (*n* = 603). A table of correlations is included in the Supplementary Materials (Supplementary Table 3). Screening measures assessed for any differences in the demographic or clinical characteristics of participants in the treatment and control groups at baseline. No significant differences were observed (*p* < 0.01). See Fig. 2 for participant flow, fidelity data characteristics, and attrition. Most participants were born in Australia (95%). They were aged between 12 and 15 years at baseline with the majority of participants being female (65.5%).

**Table 2:** Gender, age, and intolerance of uncertainty score total at baseline

		Control (*N*)	Prevention (*N*)
Gender	Female	119 (81.5%)	276 (60.4%)
	Male	27 (18.5%)	181 (39.6%)
Age	12	2 (1.4%)	37 (11.3%)
	13	47 (34.1%)	208 (63.8%)
	14	61 (44.2%)	72 (22.1%)
	15	28 (20.3%)	9 (2.8%)
Intolerance of Uncertainty	µ (SD) [80th% score]	26.73 (9.4) [36]	26.82 (8.3) [34]

### Attrition and Missing Cases

Participants were allocated to either intervention (*n* = 457) or control (*n* = 146) conditions; 53% (*n* = 317) of participants completed the first post-intervention survey, and 30% (*n* = 182) completed the third follow-up survey. To examine the suitability of missing cases replacement under a missing completely at random assumption (MCAR), all demographic and outcome variables (Table 2 and Supplementary Table 1) were examined to identify systematic patterns of missing cases, and the suitability of replacement under the assumption of missing at random (MAR; Karin et al., 2018; Little et al., 2014). These analyses identified that younger students (Wald’s *χ*^2^ = 28.93, *p* < 0.001) and school (Wald’s *χ*^2^ = 39.48, *p* < 0.001) were the only predictors of increased missing data probability, and these only accounted for a moderate amount of the total missing cases probability (Nagelkerke R Square = 8.4%). This result suggests that a Missing at Random (MAR) assumption would be suitable, provided that the replacement of missing cases was stratified by participants’ age and school. Further adjustments were incorporated to align with our design and objectives. Specifically, additional imputation parameters such as time, condition, gender, baseline scores, and treatment fidelity were included, as these factors were formally tested in our main analysis and sensitivity analyses (Woods et al., 2024).

### Analysis of Group Differences Over Time for Intolerance of Uncertainty

Table 3 displays the estimated marginal means, standard deviations, percentage of change, and 95% confidence intervals for IU across time. The table aggregates the test statistics associated with the comparison of the rate of outcome change across time between the intervention and control groups (group*time interaction). The pattern of results illustrates a significant time-by-group effect for the primary IUS outcome. The prevention condition had a significant within-group reduction in IUS over time and significantly lower post-treatment IU scores when compared to the control condition. Analyses of pre-treatment to post-treatment change for the total sample indicated statistically significant reductions for both the IU-total score (3.3%; *p*_pooled_ < 0.001), IU-Prospective (3.8%; *p*_pooled_ < 0.001) and IU Inhibitory (3.8%; *p*_pooled_ < 0.001) scores. These differences were not maintained at the third follow-up point (Table 3).

**Table 3:** ITT scores on the Intolerance of Uncertainty Scale at each time point

Outcome	Subgroup	Mean (s.d.)			Hedges g	Test statistics (*p*-value)		
		Pre-treatment	Post-treatment	Follow-up	PreTx → Post (∆%)	PreTx → PostTx	PreTx → follow-up	Po PostTx → follow-up
IUS_Total_Score	Prevention	26.821 (8.31)	25.948 (7.95)	26.955 (8.09)	3.3% (0.5 to 6)^a^	** < 0.001**	0.468	**0.001**
	Control	26.726 (9.35)	28.979 (9.74)	26.639 (8.87)	− 8.4% (− 14.3 to − 2.5)			
Inhibitory IU	Prevention	11.179 (3.77)	10.749 (3.53)	11.316 (3.59)	3.8% (1 to 6.7)	**0.003**	0.493	**0.001**
	Control	11.062 (4.54)	11.878 (4.22)	10.967 (4.06)	− 7.4% (− 13.6 to − 1.2)			
Prospective IU	Prevention	15.641 (5.1)	15.044 (4.9)	16.026 (5.02)	3.8% (0.9 to 6.7)	**0.001**	0.358	**0.0**11
	Control	15.664 (5.41)	16.761 (5.81)	15.767 (5.31)	− 7% (− 13 to − 1)			

^a^A negative numeric sign implies an increase in scores over time and a positive Δ estimate implies a reduction in scores over time

In our sampling of different school sites, variation in the number of days between baseline and post-intervention measurements occurred across the school sites. This was due to the naturalistic conditions affecting each school and their implementation of the intervention. In the control group, the average measurement window was 80 days (mean = 80.34, SD = 36.3, range = 70.37 to 97.85). In contrast, the prevention program had a longer window due to the intervention’s duration, averaging 254.4 days (SD = 51.1, range = 179.65 to 284.11). However, this variation in days did not affect the overall rate of change in the primary outcome (IUS) (Wald’s $\chi$^2^ = 0.403, *p* = 0.525) or the rate of change between groups (Wald’s $\chi$^2^ = 0.738, *p* = 0.390). Therefore the time variation was simplified to a categorical variable representing pre- and post-intervention measurements.

### Sensitivity Analyses of Condition Differences Over Time

The sensitivity analyses focused on the pre-to-post timeframe. This time frame was selected because of the higher participant retention and the finding of a main effect for the primary outcome of IU at post. In these models, the test of time x group intervention-related effects was examined while stratifying for age and gender subgroups, as well as for student subgroups who did not complete all three uncertainty lessons (to examine the effect of dosage).

The sensitivity analyses, collated in the Supplementary Tables 4 to 5, illustrate that the prevention program resulted in treatment-related mean score improvement for the primary IUS outcome, across both sexes, and across the different sampled age groups. For example, in Supplementary Table 4, the IUS model test statistics of time x group effects illustrate a significant result (*p* < 0.001), even after including a gender three-way effect. The result can be interpreted as a test of the intervention effect, even after accounting for any age or gender differences.

Finally, a dose–effect relationship was observed in the subsample of participants for whom fidelity data was collected. Within the prevention group, dosage data was obtained for 28% of participants. Of this group, the majority of students attended more than half (46.2%) or all (43.1%) of the uncertainty lessons, with some students (10.8%) attending less than half the lessons (Supplementary Table 6). At the first post-treatment timepoint, a dose effect was noticed whereby participants who received all three IU-oriented lessons showed the reduction in IU Prospective scores, whereas students who attended either half or less than half of these lessons did not show this reduction.

### Individual Rates of Reliable Change Across Groups and Subgroups of IUS Severity

To compare the rates of reliable change in children at post, an RCI cutoff of 11 points^2^ difference was the threshold for classifying the individual IUS change categories of the following: improvement, no change, or deterioration. Binary logistic regressions tested the proportion of individuals across these three categories of change. These illustrated significant group difference test rates (Wald’s *χ*^2^ = 9.998, *p* < 0.001), with more favorable rates of improved cases identified in the prevention program group (18.0%) compared to 3.2% in the control, but with similar non-significant deterioration rates (*p* = 0.459) rates between the two groups (12.3% prevention group; 9.5% in the control).

An analysis exploring RCI improvement for the top quintile, the most vulnerable subsample who were viewed to benefit most from the intervention, illustrated that the prevention program was associated with increased rates of IUS improvement for these students with higher initial IUS scores (30.1% intervention; 6.8% control). In the remainder of the sample (the lower 80% at baseline), improvement rates were also higher in the prevention group (14.3%) vs the control group (2%). Deterioration rates were also examined using the same quantile division. For those in the top quintile, there were equivalent rates of deterioration in the prevention group (5.5%) and the control group (4.1%). In the lower 80% of the sample, the rates of deterioration were not significantly different between the prevention group (14.3%) compared to the control group (11.1%, *p* = 0.478).

### Effects of the Intervention on Social Media Use and Other Secondary Outcomes

At post, two of the social media outcomes displayed a difference in the rate of change over time. The prevention program group tended to share upsetting feelings online more often and recognized the negative impact of social media on sleep, burnout, and feeling susceptible to envy, indicating that this social media use had more of an impact on them than the control group. At follow-up, four of the secondary outcomes displayed differences between the two conditions. The prevention group were less likely to engage in social media surveillance (looking at others posts), sharing upsetting feelings online, sharing life with others on social media, and texting others privately to share things that have upset them, compared to the control group. No differences were observed in FoMO with both groups reporting slight increases across the study. This suggests that the intervention did not impact Fear of Missing Out.

No other secondary outcomes showed a difference between the prevention and control group at post or follow-up, implying the prevention program was not associated with carryover effects across outcomes such as anxiety or depression (Supplementary Table 7).

Finally, the impact of the program on participants who commenced with elevated symptoms at baseline was examined (Supplementary Table 8). Importantly, the prevention program did not adversely affect any of the secondary outcomes relative to the control group in vulnerable students (*p* < 0.005).

## Discussion

The study evaluated whether the IU universal prevention program could lower Prospective IU and Inhibitory IU at post and follow-up relative to a control group. The behavioral outcome of social media use and a range of secondary outcome variables were included to determine any “secondary effects” of the intervention on adolescent wellbeing. The study also investigated the effect of the program on the most vulnerable participants who commenced the program in the top quintile on any of the psychological measures in order to establish that the prevention program (i) benefitted those who stood to gain the most from the IU intervention and (ii) did not cause harm for vulnerable individuals.

As hypothesized, the educational program led to a significant decrease on prospective IU, inhibitory IU, and total IU at post-treatment (Table 3). This effect did not vary according to age, gender, or baseline severity. Significantly, students in the top quintile demonstrated a greater reduction in their IU scores at post-treatment compared to the control students, although both showed a reduction. The rate of IU change was significantly associated with the number of lessons attended as shown with the incremental dosage effects. These results demonstrate strong support for the inference that the intervention content caused the observed changes in IU. The time by condition by baseline severity interaction showed that for all three primary outcome variables, the effect of the intervention was not materially influenced by baseline severity, further substantiating the benefits of the program at post.

While a range of in-depth theoretical reviews suggests the broader construct of IU represents a stable dispositional tendency (Carleton, 2016; Einstein, 2014; McEvoy et al., 2019), the age at which IU stabilizes has not yet been established. The percentage change in the current study showed an average decrease of 3.3% in total IU scale scores in the intervention group, while the control group increased by 8.4% in their scores. Changes were of similar magnitude on the IU Inhibitory and IU Prospective scales. These changes in the control group may reflect Hawes et al. (2021) longitudinal observation that IU scores increased between 12 and 15 years. The cognitive capacity necessary for adaptive responses to uncertainty draws on the capacity for introspective awareness and the cognitive ability to consider and predict multiple anticipated outcomes simultaneously (Osmanagaoglu et al., 2018). These capacities develop throughout adolescence, and we would expect these to be present in differing degrees within a classroom of secondary school students. The change evident in this study suggests that the intervention favorably impacted average ratings on IU Prospective (i.e., “the need to know what is going to happen”) across the course of the intervention but not at later follow-up with a significantly reduced sample of the original students. The related positive changes in awareness of social media use also provide an indication of the effectiveness of the intervention on a key behavioral indicator of IU.

While the intervention group appeared to reflect healthier social media behavior at follow-up (e.g., less surveillance, less online sharing when upset, and increased awareness of the negative effects of social media), no group differences were observed in FoMO, anxiety, or depression. Further research is required to understand whether these behaviors have clear clinical impact on mental health symptoms. In fact, their influence on mental health may be moderated by other behaviors or contexts that are concurrent (for example it may be that sharing when feeling upset online is healthy if the individual also has the support available to share feelings in person, and/or if the sharing leads to healthy reflection about the circumstance and the ability to move forward). Given FoMO can moderate the link between social media frequency and anxiety in this age group (Einstein et al., 2023), it may be essential to reduce FoMO to reduce anxiety symptoms for some students. Future studies should examine if reducing FoMO requires either individualized intervention or more systemic changes to social media access.

The fact that changes in IU beliefs were not maintained at follow-up may support the Trait Invariant component of IU described by Knowles et al. (2022). The authors suggested that the time invariant component of IU may be produced by neuroticism and that this neuroticism may result in negative beliefs about uncertainty and its implications, including the sense of uncontrollability that the individual feels when faced with uncertainty. In the current study, the changes observed at post were small (Table 4) but similar in magnitude to the effect sizes observed in other school-based universal prevention programs. Effect sizes for universal prevention programs in the literature are small, and review experts argue that the requirements for larger sample sizes to detect these effects are often impractical and expensive (Werner Seidler et al., 2017). The failure to observe a change at follow-up may have been a measurement artifact, influenced by the high level of attrition^3^, To know whether the small change at post endured at follow-up required higher retention.

**Table 4:** Effect size for within-group change and between-group change at post and follow-up (Hedges g)

Outcome	Subgroup	Pre → post	Post → follow-up	Between Group∆@Post	Between Group∆@Follow-up
IUS_Total_Score	Prevention	0.107 (− 0.022 to 0.237)	− 0.016 (− 0.146 to 0.113)	0.36 (0.172 to 0.547)	− 0.038 (− 0.224 to 0.148)
	Control	− 0.235 (− 0.466 to − 0.005)	− 0.235 (− 0.466 to − 0.005)		
IUS_Inhibitory	Prevention	0.118 (− 0.012 to 0.248)	− 0.037 (− 0.167 to 0.093)	0.304 (0.117 to 0.491)	− 0.094 (− 0.28 to 0.093)
	Control	− 0.186 (− 0.416 to 0.044)	− 0.186 (− 0.416 to 0.044)		
IUS_Prospective	Prevention	0.119 (− 0.01 to 0.249)	− 0.076 (− 0.206 to 0.054)	0.334 (0.147 to 0.521)	− 0.051 (− 0.237 to 0.135)
	Control	− 0.195 (− 0.425 to 0.035)	− 0.195 (− 0.425 to 0.035)		

To increase effectiveness and maintain gains within a naturalistic setting, it may be necessary to increase the intensity of the lessons, add maintenance lessons (Kristofferson et al., 2021), and include a whole school approach (Durlak et al., 2011) by educating parents and teachers. It is also possible that negative beliefs about IU and the subsequent tendency to catastrophize in the face of uncertainty are difficult to alter without ongoing consistent message delivery and repeated experiences in which uncertainty is activated and managed throughout schooling. In some countries, including Australia (where this study took place), concerns about adolescent mental health have led to schools trying to reduce uncertainty in academic arenas (e.g., by providing explicit instruction and providing extensive marking criteria within assessment tasks) and in social areas (e.g., providing class lists prior to the school year commencing or camp). These practices allow students and parents more anticipatory control over their experiences and reduce the need to endure uncertainty. Restricting access to social media by banning mobile devices and phones during the school day in some regions has recently come into effect (after completion of this study), and these initiatives may be helpful in learning to regulate IU or FoMO. Having young people exposed to tolerating uncertainty in safe classroom settings is likely to be an important aspect of their emotional development. This could be tested in future studies.

To date, learning to tolerate uncertainty has been associated with better outcomes in one study of children and adolescents receiving treatment for anxiety (Palitz et al., 2019). Importantly, a significant cross-cultural study of youth in high- and low- to middle-income countries reported that IU presents a significant cognitive vulnerability and called for early prevention of IU (Zemestani et al., 2022). Researchers and clinicians alike call for interventions that will assist individuals to make less threatening interpretations when faced with uncertainty (Dugas et al., 2001; Einstein & Mansell, 2016; Freeston et al., 1994; Jacoby, 2020).

### Is it Useful to Administer the Program to the Whole Cohort?

One concern about the delivery of universal prevention programs within a school context relates to the delivery of the program to a whole year group when it is possible that only a small number of children or adolescents in that group will benefit from the program.

Using a reliable change index demonstrated that a higher proportion of individuals reliably improved their IUS scores in the intervention group compared to the control group (18% vs. 3.2%), which is encouraging given the universal nature of the intervention. This intervention effect was also observed among the 20% of most symptomatic participants. Interestingly, a slightly higher proportion of participants reliably deteriorated in the education intervention (12.3%) compared to the control condition (9.5%), although this effect was relatively small and non-significant.

### Limitations

There were a number of limitations of the study brought about by the variation in program delivery times across schools, the running of the trial over several years, and the difficulty in obtaining a sizeable control group within each school. Being a naturalistic trial in which schools and teachers made school-appropriate decisions about dosage and duration, we found that lessons at School A were delivered weekly, while at School B, lessons were delivered twice a week. The school counsellor supervised the implementation of the program by teachers at School A, while the lead investigator supervised the implementation at School B. The trial was not randomized as schools selected which students would receive the intervention according to their own local programming requirements. The control group had slightly older students at baseline compared to the intervention group. The assessment time windows varied between schools according to the naturalistic conditions of each school’s curriculum and capacity to implement the intervention.

As might be expected, a range of other school-related factors influenced the implementation of the program and data collection, including the following: school programming that could not be altered, student absenteeism, and changes to timetables caused by events such as sports days and excursions. These factors could be mitigated with further training and education of the school staff about the importance of planning for specific data collection times at each stage of the intervention (emphasizing the importance of follow-up surveys) given they were scheduled to occur in a subsequent calendar year. Obstacles caused by an excess of demands when compared to intervention and school resources have been noted in other prevention studies (Barrett et al., 2006).

## Conclusion

In summary, this study examined whether changing IU in a classroom program would (1) benefit all participants, (2) have an effect on the related outcomes of social media use and secondary outcomes explored in the study, and (3) have a beneficial effect on students with elevated symptoms at baseline. We found that while the education program was of benefit to students who commenced with high IU, the benefits of the program were not maintained at follow-up for the very small sample of cases available at follow-up, and while IU changed between pre and post, and had some effect on social media use, the intervention did not have a noticeable effect on a range of secondary outcomes measured between pre and post. It is suggested that future research examine the benefits of such a program when implemented in a targeted group of adolescents and whether a briefer program with more naturalistic practice tolerating uncertainty in a range of circumstances might result in more stable or longer-term benefit for adolescents. As this study was limited to two schools that were socio-demographically similar, further research is warranted to explore how cultural and contextual factors in broader educational contexts may alter results.

## Supplementary Information

Below is the link to the electronic supplementary material.

Supplementary file1 (DOCX 185 KB)

## Data Availability

The clinical trial registration number is ACTRN12616001369415. Data is stored within the Macquarie University Repository on 10.25949/23582805.v1.

## Footnotes

1 However, not all studies demonstrating effective treatment for worry show a reduction in IU (Holmes et al., 2014).

2 Based on test–retest reliability of 0.77 observed by Khawaja and Yu (2010)

3 Unfortunately, the reduction in data at follow up, and the use of an ITT analysis, altered the results such that the pooled means at follow-up showed an increased mean on IU scores for the intervention group, whereas the mean based on an RTT sample demonstrated a drop in absolute scores. Specifically, the mean IU score for the intervention group at follow-up was 24.88 (Confidence Interval* CI *22.9 to 26.86) compared to 26.74 (*CI *25.11 to 28.37) for the control group. While at post, the mean IU score for the intervention group was 25.43 (*CI* 24.27 to 26.59) compared to 28.94 (*CI* 27.12 to 30.76) for the control group.
