# Supplementary material for: Intolerance of Uncertainty as a Central Influence on Social Media Use: A School-Based Program for Adolescents
Source: Prev Sci. 2024 Oct 25;25(8):1238–49. doi: 10.1007/s11121-024-01738-y (PMC11652582; doi:10.1007/s11121-024-01738-y)
Supplement: Supplementary file 1 — Supplementary file1 (DOCX 185 KB) [file 11121_2024_1738_MOESM1_ESM.docx]

**Supplementary Table 1**

*Scale Totals at Baseline Across Conditions*

| Scale Title | Control µ (SD) [80^th^% score] | Prevention |
| --- | --- | --- |
| Inhibitory IU | 11.06 (4.6) [17] | 11.18 (3.8) [15] |
| Prospective IU | 15.66 (5.4) [21] | 15.64 (5.1) [20] |
| CATS Social | 19.9 (8.9) [28] | 19.33 (9) [25] |
| CATS Personal | 18.12 (9.2) [26] | 17.1 (8.4) [21] |
| Anxiety (SCAS) | 31.75 (17) [49] | 29.67 (16) [41] |
| Urgency | 27.55 (7.1) [35] | 31.22 (7) [38] |
| Envy | 18.85 (7.5) [27] | 18.35 (7.3) [26] |
| GAD (SCAS) | 7.34 (3.8) [12] | 6.72 (3.6) [10] |
| OCD (SCAS) | 5.25 (3.9) [10] | 5.09 (3.4) [8] |
| Social Anxiety (SCAS) | 7.94 (3.6) [12] | 7.24 (3.6) [11] |
| Experiential avoidance (AFQ) | 37.65 (12.7) [51] | 35.74 (12) [45] |
| Distress disclosure (DDI) | 42.1 (12.3) [54] | 42.34 (12) [53] |
| Rumination (RTQ) | 25.16 (10.2) [35] | 23.92 (9.5) [33] |
| Brief Fear of Negative Evaluation (BFNE) | 19.69 (8.5) [29] | 18.63 (8.2) [25] |
| Positive coping with Pos coping Anger | 17.89 (4.1) [23] | 17.87 (4.1) [21] |
| SCS Total | 3.13 (0.6) [4] | 3.14 (0.6) [4] |
| Depression SMFQ | 6.54 (5.7) [12] | 6.11 (5.6) [11] |
| Public Sharing* | 6.67 (2) [9] | 6.47 (2.5) [9] |
| Surveillance_SM* | 11.97 (4) [16] | 11.42 (5.2) [16] |
| Upset share online* | 1.85 (1) [4] | 1.64 (1.1) [3] |
| Text priv* | 2.06 (1.1) [4] | 2.17 (1.3) [4] |
| SM Reduction* | 1.94 (1.2) [4] | 2.1 (1.1) [4] |
| FOMO* | 4.7 (2) [7] | 4.15 (2) [6] |
| Neg_eff_SM* | 3.12 (2.8) [7] | 2.66 (2.8) [5] |
| Mean acts online* | 4.21 (2.2) [6] | 4.37 (2.2) [6] |
| Self Kindness SCS | 6.15 (1.5) [8] | 5.77 (1.9) [8] |
| Self-Judgment SCS | 6.48 (2.3) [10] | 6.96 (2.2) [10] |
| Common Humanity SCS | 5.84 (1.8) [8] | 5.26 (1.9) [8] |
| Isolation SCS | 6.23 (2.1) [10] | 6.75 (2.1) [9] |
| Mindfulness SCS | 6.39 (1.8) [9] | 6.08 (1.9) [9] |
| Over-identification SCS | 6.26 (1.4) [8] | 6.3 (1.5) [8] |
| Substance Use | 2.34 (1) [0] | 2.34 (1.1) [0] |

| IU Intolerance of Uncertainty; CATS Children’s Automatic Thoughts Scale; SCAS Spence Children’s Anxiety Scale SCS Self Compassion Scale Surveillance SM: Surveillance use of social media; Text priv: Texting friends privately when upset SM Reduction Social Media Reduction; FOMO: Fear of Missing out if not connected online; Neg_Eff_SM Negative Effects of social media. For a breakdown of social media items included in each *scale, see Supplementary Table 7. |
| --- |

**Supplementary Table 2**

*Secondary Outcome Measures*

| Factor | Scale | Number of items | Likert scale Responses | Internal consistency established in age | Authors | Item example |
| --- | --- | --- | --- | --- | --- | --- |
| Catastrophising and Personalising | The Children’s Automatic Thoughts Scale (CATS) | 5 | 5-point | Yes | Schniering & Rapee, 2002 | “Kids are going to laugh at me”  “I can’t do anything right” |
| Anxiety | The Spence’s Children’s Anxiety Scale (SCAS) | 44 | 4-point | Yes | Spence, 1998 | “All of a sudden I feel really scared for no reason at all” |
| Urgency | UPPS Impulsive Behaviour Scale | 12 | 4-point | Yes | Whiteside & Lynam, 2001 | “It is hard for me to resist acting on feelings” |
| Envy | Dispositional Envy Scale (DES) | 8 | 5-point | Yes | Smith et al, 1999 | “It is so frustrating to see some people succeed so easily” |
| Experiential Avoidance | Avoidance and Fusion Questionnaire for Youth (AFQ-Y) | 17 | 5-point | Yes | Greco et al., 2008 | “I push away thoughts and feelings that I don’t like” |
| Distress when disclosing personal information | Distress Disclosure Index (DDI) | 12 | 5-point | Yes | Kahn & Hessling, 2001 | “When I feel upset I usually confide in my friends”  “I prefer not to talk about my problems” |
| Rumination | Repetitive Thinking Questionnaire-10 (RTQ-10) | 10 | 5-point | Yes | McEvoy et al., 2014; McEvoy et al., 2019 | “Once you start thinking about the situation, you can’t stop” |
| Fear of Negative Evaluation | The Brief Fear of Negative Evaluation Scale, Straightforward Items (BFNE-S) | 8 | 4-point | Yes | Rodebaugh et al., 2004 | “I am frequently afraid of other people noticing my shortcomings” |
| Anger | Positive Coping with Anger Subscale of the Multidimensional School Anger Inventory | 8 | 4-point | Yes | Furlong et al. 2002 | “When I get angry, I think about something else.” |
| Self-Compassion | The Self-Compassion Scale Short Form (SCS-SF) | 12 | 5-point | Yes | Raes et al., 2011 | I try to see my failings as part of the human condition. |
| Depression | Short Mood and Feelings Questionnaire (SMFQ) | 13 | 3-point | Yes | Angold et al., 1995 | I feel miserable or unhappy |
| Social Media | Drawn from 3 scales. i)Australian adult and adolescent sample using the Australian Psychological Society Stress and Wellbeing Survey assessed whether participants experience discomfort and a fear of missing out when disconnected from social media. ii) style of social media use; iii) Fear of Missing Out | 19 | 5-8 point | No | i) Australian Psychological Society, 2015. ii)Tandoc et al., 2015 iii)Przybylski et al. (2013). | Items combined into subscales as set out in Supplementary Table 8. |
| Peer influence of substance use | Patterns of Alcohol Use measure from SHAHRP | 2 | 5-point | No* | McBride, Farringdon & Midford, 2000 | “Substances (alcohol, drugs, medication) are the immediate way I respond to my thoughts about a situation when I feel distressed or upset.” |

*Two items used from original scale

**Supplementary Table 3**

*Table of Correlations*

|  | IUS_Total | Inhibitory IU | Prospective IU | CATS_Social | | CATS_Personal | | SCAS | Urgency | | Envy | | GAD | | OCD | SA | | AFQ | | DDI | RTQ | | BFNE | | | Pos coping Anger | | SCS Total |
| --- | --- | --- | --- | --- | --- | --- | --- | --- | --- | --- | --- | --- | --- | --- | --- | --- | --- | --- | --- | --- | --- | --- | --- | --- | --- | --- | --- | --- |
| IUS Total | 1 | .916** | .952** | .434** | | .420** | | .633** | .080* | | .522** | | .577** | | .566** | .527** | | .622** | | -.170** | .470** | | .511** | | | 0.048 | | -.379** |
| Inhibitory IU | .916** | 1 | .750** | .382** | | .369** | | .569** | 0.079 | | .472** | | .489** | | .526** | .481** | | .569** | | -.155** | .393** | | .439** | | | 0.043 | | -.341** |
| Prospective IU | .952** | .750** | 1 | .425** | | .411** | | .610** | 0.072 | | .502** | | .580** | | .534** | .502** | | .594** | | -.163** | .476** | | .509** | | | 0.047 | | -.366** |
| CATS Social_ | .434** | .382** | .425** | 1 | | .727** | | .608** | .117** | | .601** | | .564** | | .436** | .667** | | .654** | | -.233** | .578** | | .781** | | | -0.012 | | -.497** |
| CATS Personal modified | .420** | .369** | .411** | .727** | | 1 | | .590** | 0.077 | | .619** | | .573** | | .446** | .543** | | .712** | | -.371** | .638** | | .665** | | | -0.076 | | -.622** |
| SCAS | .633** | .569** | .610** | .608** | | .590** | | 1 | .110** | | .553** | | .875** | | .800** | .788** | | .695** | | -.152** | .581** | | .622** | | | 0.071 | | -.450** |
| Urgency | .080* | 0.079 | 0.072 | .117** | | 0.077 | | .110** | 1 | | 0.042 | | .084* | | .113** | .088* | | .092* | | -0.034 | 0.069 | | .131** | | | 0.004 | | -0.072 |
| Envy | .522** | .472** | .502** | .601** | | .619** | | .553** | 0.042 | | 1 | | .513** | | .448** | .537** | | .606** | | -.258** | .547** | | .606** | | | -0.058 | | -.575** |
| GAD SCAS | .577** | .489** | .580** | .564** | | .573** | | .875** | .084* | | .513** | | 1 | | .622** | .644** | | .643** | | -.156** | .573** | | .610** | | | .088* | | -.426** |
| OCD SCAS | .566** | .526** | .534** | .436** | | .446** | | .800** | .113** | | .448** | | .622** | | 1 | .562** | | .576** | | -.155** | .504** | | .461** | | | 0.067 | | -.363** |
| SA SCAS | .527** | .481** | .502** | .667** | | .543** | | .788** | .088* | | .537** | | .644** | | .562** | 1 | | .615** | | -.170** | .496** | | .648** | | | 0.014 | | -.458** |
| AFQ | .622** | .569** | .594** | .654** | | .712** | | .695** | .092* | | .606** | | .643** | | .576** | .615** | | 1 | | -.276** | .688** | | .712** | | | 0.031 | | -.543** |
| DDI | -.170** | -.155** | -.163** | -.233** | | -.371** | | -.152** | -0.034 | | -.258** | | -.156** | | -.155** | -.170** | | -.276** | | 1 | -.251** | | -.242** | | | .376** | | .445** |
| RTQ | .470** | .393** | .476** | .578** | | .638** | | .581** | 0.069 | | .547** | | .573** | | .504** | .496** | | .688** | | -.251** | 1 | | .721** | | | 0.077 | | -.552** |
| BFNE | .511** | .439** | .509** | .781** | | .665** | | .622** | .131** | | .606** | | .610** | | .461** | .648** | | .712** | | -.242** | .721** | | 1 | | | 0.047 | | -.573** |
| Pos coping Anger | 0.048 | 0.043 | 0.047 | -0.012 | | -0.076 | | 0.071 | 0.004 | | -0.058 | | .088* | | 0.067 | 0.014 | | 0.031 | | .376** | 0.077 | | 0.047 | | | 1 | | .303** |
| SCS Total | -.379** | -.341** | -.366** | -.497** | | -.622** | | -.450** | -0.072 | | -.575** | | -.426** | | -.363** | -.458** | | -.543** | | .445** | -.552** | | -.573** | | | .303** | | 1 |
| SMFQ | .415** | .350** | .418** | .671** | | .828** | | .605** | 0.058 | | .602** | | .593** | | .455** | .546** | | .706** | | -.329** | .686** | | .679** | | | -0.024 | | -.604** |
| Pub_Share | .098* | .096* | .088* | .186** | | .143** | | .208** | 0.024 | | .153** | | .181** | | .177** | .189** | | .208** | | -0.005 | .119** | | .153** | | | -0.007 | | -.131** |
| Surveillance_SM | .082* | .084* | 0.072 | .196** | | .123** | | .154** | 0.002 | | .167** | | .146** | | .101* | .162** | | .161** | | -0.036 | .126** | | .160** | | | 0.003 | | -.151** |
| Upset share online | .136** | .123** | .131** | .135** | | .118** | | .181** | 0.041 | | .107** | | .168** | | .101* | .135** | | .170** | | 0.006 | .081* | | .110** | | | 0.027 | | -.108** |
| SM Reduction | .200** | .175** | .197** | .176** | | .158** | | .241** | 0.022 | | .324** | | .217** | | .209** | .156** | | .189** | | -0.053 | .185** | | .182** | | | 0.057 | | -.161** |
| FOMO | .302** | .269** | .293** | .310** | | .289** | | .390** | 0.016 | | .326** | | .366** | | .277** | .369** | | .401** | | -0.072 | .346** | | .357** | | | 0.041 | | -.271** |
| Neg_eff_SM | .281** | .243** | .278** | .314** | | .283** | | .360** | 0.016 | | .402** | | .322** | | .281** | .309** | | .329** | | -.123** | .256** | | .318** | | | -0.036 | | -.299** |
| Mean acts online | .208** | .192** | .197** | .128** | | .152** | | .219** | 0.033 | | .325** | | .164** | | .249** | .108** | | .135** | | -.123** | .144** | | .128** | | | -.135** | | -.201** |
| Textpriv | 0.036 | 0.042 | 0.027 | .199** | | .163** | | .184** | .110** | | .157** | | .154** | | .096* | .179** | | .159** | | 0.045 | .161** | | .149** | | | -0.013 | | -.158** |
| SCS_SK | -0.003 | -0.034 | 0.021 | -0.066 | | -.172** | | -0.026 | -0.024 | | -.117** | | -0.009 | | -0.052 | -0.038 | | -0.033 | | .324** | -0.018 | | -0.016 | | | .413** | | .554** |
| SCS_SJ | -.419** | -.350** | -.427** | -.538** | | -.609** | | -.465** | -0.070 | | -.566** | | -.456** | | -.355** | -.493** | | -.594** | | .296** | -.640** | | -.663** | | | -0.019 | | .703** |
| Common Humanity | 0.075 | 0.032 | .100* | .089* | | 0.002 | | 0.064 | 0.015 | | 0.043 | | .082* | | 0.045 | 0.050 | | .091* | | .186** | .150** | | .134** | | | .355** | | .344** |
| Isolation | -.397** | -.333** | -.402** | -.554** | | -.569** | | -.501** | -0.067 | | -.568** | | -.467** | | -.379** | -.517** | | -.611** | | .261** | -.646** | | -.661** | | | -0.033 | | .634** |
| Mindfulness | -0.021 | -0.052 | 0.005 | -0.021 | | -.115** | | -0.013 | 0.002 | | -.121** | | 0.003 | | -0.017 | 0.007 | | -0.014 | | .249** | 0.037 | | 0.034 | | | .462** | | .544** |
| Overidentification | -.279** | -.244** | -.275** | -.317** | | -.426** | | -.322** | -.088* | | -.433** | | -.325** | | -.275** | -.287** | | -.362** | | .266** | -.398** | | -.412** | | | .208** | | .740** |
| Substance | .084* | 0.051 | .100* | 0.071 | | .112** | | .124** | 0.071 | | .088* | | .108** | | .128** | 0.076 | | .179** | | -.105* | 0.074 | | 0.051 | | | -.179** | | -.139** |
| **. Correlation is significant at the 0.01 level (2-tailed). | | | | | | |  | | |  | |  | |  | | |  | |  |  | |  | | |  | |  | |
| *. Correlation is significant at the 0.05 level (2-tailed). | | | | |  | |  | | |  | |  | |  | | |  | |  |  | |  | | |  | |  | |
|  | SMFQ | Pub_Share | Surveillance_SM | Upsetshare | | Insight_SM | | FOMO | Neg_eff_SM | | Mean acts online | | Textpriv | | SCS_SK | SCS_SJ | | Common Humanity | | Isolation | Mindfulness | | | Overidentification | | Substance | |  |
| IUS_Total | .415** | .098* | .082* | .136** | | .200** | | .302** | .281** | | .208** | | 0.036 | | -0.003 | -.419** | | 0.075 | | -.397** | -0.021 | | | -.279** | | .084* | |  |
| Inhibitory IU | .350** | .096* | .084* | .123** | | .175** | | .269** | .243** | | .192** | | 0.042 | | -0.034 | -.350** | | 0.032 | | -.333** | -0.052 | | | -.244** | | 0.051 | |  |
| Prospective IU | .418** | .088* | 0.072 | .131** | | .197** | | .293** | .278** | | .197** | | 0.027 | | 0.021 | -.427** | | .100* | | -.402** | 0.005 | | | -.275** | | .100* | |  |
| CATS_Social | .671** | .186** | .196** | .135** | | .176** | | .310** | .314** | | .128** | | .199** | | -0.066 | -.538** | | .089* | | -.554** | -0.021 | | | -.317** | | 0.071 | |  |
| CATS_Personal_l | .828** | .143** | .123** | .118** | | .158** | | .289** | .283** | | .152** | | .163** | | -.172** | -.609** | | 0.002 | | -.569** | -.115** | | | -.426** | | .112** | |  |
| SCAS_TOTAL | .605** | .208** | .154** | .181** | | .241** | | .390** | .360** | | .219** | | .184** | | -0.026 | -.465** | | 0.064 | | -.501** | -0.013 | | | -.322** | | .124** | |  |
| Urgency | 0.058 | 0.024 | 0.002 | 0.041 | | 0.022 | | 0.016 | 0.016 | | 0.033 | | .110** | | -0.024 | -0.070 | | 0.015 | | -0.067 | 0.002 | | | -.088* | | 0.071 | |  |
| Envy | .602** | .153** | .167** | .107** | | .324** | | .326** | .402** | | .325** | | .157** | | -.117** | -.566** | | 0.043 | | -.568** | -.121** | | | -.433** | | .088* | |  |
| SCAS_GAD | .593** | .181** | .146** | .168** | | .217** | | .366** | .322** | | .164** | | .154** | | -0.009 | -.456** | | .082* | | -.467** | 0.003 | | | -.325** | | .108** | |  |
| SCAS_OCD | .455** | .177** | .101* | .101* | | .209** | | .277** | .281** | | .249** | | .096* | | -0.052 | -.355** | | 0.045 | | -.379** | -0.017 | | | -.275** | | .128** | |  |
| SCAS_SA | .546** | .189** | .162** | .135** | | .156** | | .369** | .309** | | .108** | | .179** | | -0.038 | -.493** | | 0.050 | | -.517** | 0.007 | | | -.287** | | 0.076 | |  |
| AFQ | .706** | .208** | .161** | .170** | | .189** | | .401** | .329** | | .135** | | .159** | | -0.033 | -.594** | | .091* | | -.611** | -0.014 | | | -.362** | | .179** | |  |
| DDI | -.329** | -0.005 | -0.036 | 0.006 | | -0.053 | | -0.072 | -.123** | | -.123** | | 0.045 | | .324** | .296** | | .186** | | .261** | .249** | | | .266** | | -.105* | |  |
| RTQ | .686** | .119** | .126** | .081* | | .185** | | .346** | .256** | | .144** | | .161** | | -0.018 | -.640** | | .150** | | -.646** | 0.037 | | | -.398** | | 0.074 | |  |
| BFNE | .679** | .153** | .160** | .110** | | .182** | | .357** | .318** | | .128** | | .149** | | -0.016 | -.663** | | .134** | | -.661** | 0.034 | | | -.412** | | 0.051 | |  |
| Pos coping Anger | -0.024 | -0.007 | 0.003 | 0.027 | | 0.057 | | 0.041 | -0.036 | | -.135** | | -0.013 | | .413** | -0.019 | | .355** | | -0.033 | .462** | | | .208** | | -.179** | |  |
| SCS Total | -.604** | -.131** | -.151** | -.108** | | -.161** | | -.271** | -.299** | | -.201** | | -.158** | | .554** | .703** | | .344** | | .634** | .544** | | | .740** | | -.139** | |  |
| SMFQ | 1 | .186** | .155** | .114** | | .180** | | .361** | .334** | | .137** | | .147** | | -.106** | -.620** | | 0.070 | | -.611** | -.093* | | | -.418** | | .130** | |  |
| Pulic Sharing | .186** | 1 | .711** | .472** | | -.108** | | .556** | .350** | | -0.035 | | .384** | | -0.068 | -0.077 | | 0.034 | | -.109** | -.112** | | | -0.076 | | .164** | |  |
| Surveillance_SM | .155** | .711** | 1 | .444** | | -.136** | | .488** | .271** | | -.082* | | .435** | | -0.078 | -.121** | | 0.006 | | -.092* | -.094* | | | -.091* | | .097* | |  |
| Upset share online | .114** | .472** | .444** | 1 | | -0.006 | | .340** | .224** | | 0.024 | | .305** | | -0.038 | -.087* | | 0.044 | | -.086* | -.121** | | | -0.077 | | .124** | |  |
| SM Reduction | .180** | -.108** | -.136** | -0.006 | | 1 | | -0.061 | .202** | | .355** | | 0.030 | | 0.054 | -.201** | | 0.005 | | -.190** | -0.002 | | | -0.071 | | 0.011 | |  |
| FOMO | .361** | .556** | .488** | .340** | | -0.061 | | 1 | .473** | | .104* | | .265** | | -0.026 | -.305** | | .118** | | -.307** | -0.052 | | | -.195** | | .183** | |  |
| Neg_eff_SM | .334** | .350** | .271** | .224** | | .202** | | .473** | 1 | | .232** | | .195** | | -.094* | -.262** | | 0.006 | | -.268** | -.136** | | | -.214** | | .186** | |  |
| Mean acts online | .137** | -0.035 | -.082* | 0.024 | | .355** | | .104* | .232** | | 1 | | 0.029 | | -.117** | -.145** | | -0.047 | | -.113** | -.160** | | | -.181** | | .113** | |  |
| Textpriv | .147** | .384** | .435** | .305** | | 0.030 | | .265** | .195** | | 0.029 | | 1 | | -.097* | -.110** | | -0.025 | | -.124** | -.089* | | | -.119** | | .083* | |  |
| SK^1^ | -.106** | -0.068 | -0.078 | -0.038 | | 0.054 | | -0.026 | -.094* | | -.117** | | -.097* | | 1 | 0.043 | | .542** | | -.088* | .681** | | | .544** | | -.112** | |  |
| SJ | -.620** | -0.077 | -.121** | -.087* | | -.201** | | -.305** | -.262** | | -.145** | | -.110** | | 0.043 | 1 | | -.234** | | .685** | 0.017 | | | .548** | | -0.069 | |  |
| Common Humanity | 0.070 | 0.034 | 0.006 | 0.044 | | 0.005 | | .118** | 0.006 | | -0.047 | | -0.025 | | .542** | -.234** | | 1 | | -.214** | .508** | | | .090* | | -0.050 | |  |
| Isolation | -.611** | -.109** | -.092* | -.086* | | -.190** | | -.307** | -.268** | | -.113** | | -.124** | | -.088* | .685** | | -.214** | | 1 | -.097* | | | .406** | | -0.054 | |  |
| Mindfulness | -.093* | -.112** | -.094* | -.121** | | -0.002 | | -0.052 | -.136** | | -.160** | | -.089* | | .681** | 0.017 | | .508** | | -.097* | 1 | | | .362** | | -.136** | |  |
| Overidentification | -.418** | -0.076 | -.091* | -0.077 | | -0.071 | | -.195** | -.214** | | -.181** | | -.119** | | .544** | .548** | | .090* | | .406** | .362** | | | 1 | | -.126** | |  |
| Substance | .130** | .164** | .097* | .124** | | 0.011 | | .183** | .186** | | .113** | | .083* | | -.112** | -0.069 | | -0.050 | | -0.054 | -.136** | | | -.126** | | 1 | |  |
| **. Correlation is significant at the 0.01 level (2-tailed). | | | | | | | | | |  | |  | |  | | |  | |  |  | |  | | |  | |  | |
| *. Correlation is significant at the 0.05 level (2-tailed). | | | | | | |  | | |  | |  | |  | | |  | |  |  | |  | | |  | |  | |

^1^Six subscales listed above from the SCS are Self Kindness (SK), Self Judgment (SJ), common humanity, isolation, mindfulness and overidentification

| **Supplementary Table 4**  *Sensitivity Analyses for the Effects of Gender; Considering Time (Pre to Post), Condition and Simplified Gender Effects* | | | | | | | | |
| --- | --- | --- | --- | --- | --- | --- | --- | --- |
| Outcome variable | Condition | Gender | Pre-treatment (SD) | Post-treatment (SD) | Sample (n) | Time*Condition  (p-value) | Time*gender  (p-value) | Time*Condition*gender  (p-value) |
| IUS Total | Int | Female | 28.062 (8.55) | 26.885 (7.98) | 276 | ***p<0.001*** | *p=0.56* | *p=0.547* |
|  | Cont | Female | 27.16 (9.33) | 29.18 (9.8) | 119 |  |  |  |
|  | Int | Male | 24.928 (7.54) | 24.518 (7.69) | 181 |  |  |  |
|  | Cont | Male | 24.815 (9.21) | 28.094 (9.47) | 27 |  |  |  |
| Inhibitory IU | Int | Female | 11.63 (3.86) | 11.168 (3.6) | 276 | ***p=0.004*** | *p=0.709* | *p=0.752* |
|  | Cont | Female | 11.37 (4.53) | 12.057 (4.26) | 119 |  |  |  |
|  | Int | Male | 10.492 (3.53) | 10.11 (3.32) | 181 |  |  |  |
|  | Cont | Male | 9.704 (4.33) | 11.088 (3.95) | 27 |  |  |  |
| Prospective IU | Int | Female | 16.431 (5.24) | 15.636 (4.95) | 276 | ***P<0.001*** | *p=0.577* | *p=0.225* |
|  | Cont | Female | 15.79 (5.39) | 16.812 (5.83) | 119 |  |  |  |
|  | Int | Male | 14.436 (4.63) | 14.141 (4.68) | 181 |  |  |  |
|  | Cont | Male | 15.111 (5.49) | 16.536 (5.72) | 27 |  |  |  |
| CATS Social | Int | Female | 21.413 (9.38) | 22.677 (9.65) | 276 | *p=0.746* | *p=0.64* | *p=0.629* |
|  | Cont | Female | 20.414 (9.24) | 21.924 (8.63) | 119 |  |  |  |
|  | Int | Male | 16.149 (7.21) | 18.511 (7.45) | 181 |  |  |  |
|  | Cont | Male | 17.667 (6.67) | 17.779 (6.25) | 27 |  |  |  |
| CATS Personal | Int | Female | 18.692 (8.8) | 22.113 (9.54) | 276 | *p=0.514* | *p=0.007* | *p=0.416* |
|  | Cont | Female | 18.224 (9.33) | 21.706 (9) | 119 |  |  |  |
|  | Int | Male | 14.696 (6.98) | 17.895 (6.97) | 181 |  |  |  |
|  | Cont | Male | 17.741 (8.61) | 18.195 (8.34) | 27 |  |  |  |
| SCAS | Int | Female | 34.101 (15.98) | 33.171 (17.34) | 276 | *p=0.588* | *p=0.628* | *p=0.721* |
|  | Cont | Female | 34.034 (17.03) | 33.534 (15.75) | 119 |  |  |  |
|  | Int | Male | 22.912 (13.53) | 23.59 (13.85) | 181 |  |  |  |
|  | Cont | Male | 21.704 (12.57) | 24.404 (14.1) | 27 |  |  |  |
| Urgency | Int | Female | 30.971 (6.36) | 29.989 (7.4) | 276 | *p=0.609* | *p=0.969* | *p=0.015* |
|  | Cont | Female | 27.857 (7.3) | 28.331 (6.59) | 119 |  |  |  |
|  | Int | Male | 31.597 (7.81) | 32.286 (7.22) | 181 |  |  |  |
|  | Cont | Male | 26.185 (6.02) | 25.993 (6.89) | 27 |  |  |  |
| Envy | Int | Female | 20 (7.4) | 20.271 (6.79) | 276 | *p=0.975* | *p=0.256* | *p=0.076* |
|  | Cont | Female | 19.022 (7.52) | 20.345 (7.51) | 119 |  |  |  |
|  | Int | Male | 15.829 (6.43) | 16.253 (6.03) | 181 |  |  |  |
|  | Cont | Male | 18.333 (7.56) | 16.547 (6.26) | 27 |  |  |  |
| GAD SCAS | Int | Female | 7.652 (3.52) | 7.556 (3.75) | 276 | *p=0.528* | *p=0.46* | *p=0.678* |
|  | Cont | Female | 7.723 (3.9) | 7.333 (3.39) | 119 |  |  |  |
|  | Int | Male | 5.287 (3.15) | 5.182 (2.99) | 181 |  |  |  |
|  | Cont | Male | 5.667 (2.98) | 5.346 (2.97) | 27 |  |  |  |
| OCD SCAS | Int | Female | 5.554 (3.42) | 5.354 (3.62) | 276 | *p=0.782* | *p=0.955* | *p=0.557* |
|  | Cont | Female | 5.555 (3.99) | 5.029 (3.15) | 119 |  |  |  |
|  | Int | Male | 4.376 (3.16) | 4.325 (2.82) | 181 |  |  |  |
|  | Cont | Male | 3.926 (3.11) | 4.426 (2.98) | 27 |  |  |  |
| SA SCAS | Int | Female | 8.174 (3.46) | 7.971 (3.58) | 276 | *p=0.406* | *p=0.69* | *p=0.967* |
|  | Cont | Female | 8.378 (3.66) | 7.88 (3.53) | 119 |  |  |  |
|  | Int | Male | 5.823 (3.35) | 6.094 (3.49) | 181 |  |  |  |
|  | Cont | Male | 6 (2.43) | 5.891 (3.16) | 27 |  |  |  |
| AFQ | Int | Female | 38.025 (11.85) | 39.81 (13.4) | 276 | *p=0.626* | *p=0.206* | *p=0.534* |
|  | Cont | Female | 38.334 (12.8) | 41.994 (12.19) | 119 |  |  |  |
|  | Int | Male | 32.325 (11.49) | 32.675 (11) | 181 |  |  |  |
|  | Cont | Male | 34.333 (11.53) | 36.257 (10.59) | 27 |  |  |  |
| DDI | Int | Female | 42.598 (12.78) | 42.367 (11.74) | 276 | *p=0.235* | *p=0.139* | *p=0.958* |
|  | Cont | Female | 42.27 (12.53) | 42.788 (11.36) | 119 |  |  |  |
|  | Int | Male | 41.986 (10.69) | 40.191 (10.58) | 181 |  |  |  |
|  | Cont | Male | 41.074 (10.79) | 39.447 (12.19) | 27 |  |  |  |
| RTQ | Int | Female | 26.254 (9.57) | 26.552 (9) | 276 | *p=0.156* | *p=0.153* | *p=0.014* |
|  | Cont | Female | 25.384 (10.36) | 28.129 (9.23) | 119 |  |  |  |
|  | Int | Male | 20.305 (8.17) | 23.58 (8.53) | 181 |  |  |  |
|  | Cont | Male | 24.593 (9.24) | 29.821 (8.5) | 27 |  |  |  |
| BFNE | Int | Female | 20.993 (8.12) | 21.722 (7.88) | 276 | *p=0.474* | *p=0.056* | *p=0.066* |
|  | Cont | Female | 20.17 (8.46) | 21.376 (6.93) | 119 |  |  |  |
|  | Int | Male | 15.042 (6.95) | 18.209 (6.97) | 181 |  |  |  |
|  | Cont | Male | 17.667 (8.17) | 20.424 (6.49) | 27 |  |  |  |
| Pos coping Anger | Int | Female | 18.489 (3.64) | 18.533 (4.12) | 276 | *p=0.836* | *p=0.883* | *p=0.175* |
|  | Cont | Female | 18.422 (3.73) | 18.361 (4.1) | 119 |  |  |  |
|  | Int | Male | 16.931 (4.52) | 16.598 (4.04) | 181 |  |  |  |
|  | Cont | Male | 15.519 (4.79) | 15.675 (3.36) | 27 |  |  |  |
| SCS | Int | Female | 3.061 (0.61) | 2.953 (0.55) | 276 | *p=0.674* | *p=0.361* | *p=0.085* |
|  | Cont | Female | 3.119 (0.63) | 3.057 (0.68) | 119 |  |  |  |
|  | Int | Male | 3.261 (0.52) | 3.117 (0.54) | 181 |  |  |  |
|  | Cont | Male | 3.155 (0.52) | 2.973 (0.53) | 27 |  |  |  |
| SMFQ | Int | Female | 7.345 (5.8) | 9.537 (6.34) | 276 | *p=0.135* | *p=0.557* | *p=0.066* |
|  | Cont | Female | 6.724 (5.68) | 8.559 (6.06) | 119 |  |  |  |
|  | Int | Male | 4.272 (4.6) | 6.37 (5.03) | 181 |  |  |  |
|  | Cont | Male | 5.963 (5.76) | 7.703 (4.95) | 27 |  |  |  |
| Public Sharing | Int | Female | 6.708 (2.22) | 6.692 (2.38) | 276 | *p=0.568* | *p=0.184* | *p=0.159* |
|  | Cont | Female | 6.939 (1.85) | 6.791 (2.11) | 119 |  |  |  |
|  | Int | Male | 6.108 (2.8) | 6.196 (2.53) | 181 |  |  |  |
|  | Cont | Male | 5.37 (2.08) | 5.674 (2.03) | 27 |  |  |  |
| Surveillance SM | Int | Female | 12.122 (4.53) | 12.546 (5.08) | 276 | *p=0.053* | *p=0.313* | *p=0.21* |
|  | Cont | Female | 12.592 (3.67) | 12.087 (4.16) | 119 |  |  |  |
|  | Int | Male | 10.341 (5.78) | 11.678 (4.99) | 181 |  |  |  |
|  | Cont | Male | 9.259 (4.17) | 9.961 (4.41) | 27 |  |  |  |
| Upset share online | Int | Female | 1.793 (1) | 1.711 (1.09) | 276 | *p=0.374* | *p=0* | *p=0.126* |
|  | Cont | Female | 2 (1.01) | 1.573 (0.82) | 119 |  |  |  |
|  | Int | Male | 1.399 (1.13) | 1.767 (1.08) | 181 |  |  |  |
|  | Cont | Male | 1.148 (0.36) | 1.454 (0.78) | 27 |  |  |  |
| Textpriv | Int | Female | 2.31 (1.25) | 3.003 (1.51) | 276 | *p=0.756* | *p=0.075* | *p=0.025* |
|  | Cont | Female | 2.048 (1.14) | 2.753 (1.11) | 119 |  |  |  |
|  | Int | Male | 1.95 (1.31) | 2.463 (1.54) | 181 |  |  |  |
|  | Cont | Male | 2.185 (0.91) | 2.524 (1.01) | 27 |  |  |  |
| SM Reduction | Int | Female | 2.214 (1.08) | 2.181 (1.11) | 276 | *p=0.888* | *p=0.804* | *p=0.74* |
|  | Cont | Female | 1.962 (1.18) | 2.056 (1.08) | 119 |  |  |  |
|  | Int | Male | 1.923 (1.07) | 1.917 (1.08) | 181 |  |  |  |
|  | Cont | Male | 1.852 (0.97) | 1.892 (0.92) | 27 |  |  |  |
| FOMO | Int | Female | 4.526 (2.04) | 4.853 (2.04) | 276 | *p=0.858* | *p=0.029* | *p=0.907* |
|  | Cont | Female | 4.925 (1.98) | 4.879 (2.25) | 119 |  |  |  |
|  | Int | Male | 3.564 (1.88) | 4.187 (2.1) | 181 |  |  |  |
|  | Cont | Male | 3.704 (1.56) | 4.602 (1.95) | 27 |  |  |  |
| Neg eff SM | Int | Female | 2.995 (2.85) | 3.915 (2.72) | 276 | *p=0.133* | *p=0.114* | *p=0.628* |
|  | Cont | Female | 3.442 (2.86) | 3.192 (2.68) | 119 |  |  |  |
|  | Int | Male | 2.164 (2.58) | 3.126 (2.49) | 181 |  |  |  |
|  | Cont | Male | 1.704 (2.11) | 2.488 (2.27) | 27 |  |  |  |
| Mean acts online | Int | Female | 4.359 (2.18) | 4.885 (2.15) | 276 | *p=0.238* | *p=0.377* | *p=0.527* |
|  | Cont | Female | 4.176 (2.12) | 4.812 (2.41) | 119 |  |  |  |
|  | Int | Male | 4.392 (2.15) | 5.171 (2.05) | 181 |  |  |  |
|  | Cont | Male | 4.37 (2.27) | 4.488 (1.7) | 27 |  |  |  |
| SCS SK | Int | Female | 6.036 (1.69) | 5.546 (1.68) | 276 | *p=0.602* | *p=0.932* | *p=0.155* |
|  | Cont | Female | 6.196 (1.51) | 5.829 (1.74) | 119 |  |  |  |
|  | Int | Male | 5.365 (1.99) | 4.947 (1.78) | 181 |  |  |  |
|  | Cont | Male | 6.025 (1.71) | 5.66 (1.67) | 27 |  |  |  |
| SCS SJ | Int | Female | 6.384 (2.12) | 6.22 (1.98) | 276 | *p=0.563* | *p=0.09* | *p=0.037* |
|  | Cont | Female | 6.419 (2.27) | 6.34 (2.35) | 119 |  |  |  |
|  | Int | Male | 7.84 (1.95) | 7.069 (1.83) | 181 |  |  |  |
|  | Cont | Male | 6.736 (2) | 6.253 (1.97) | 27 |  |  |  |
| Common Humanity | Int | Female | 5.499 (1.85) | 5.471 (1.68) | 276 | *p=0.351* | *p=0.516* | *p=0.466* |
|  | Cont | Female | 5.872 (1.72) | 5.779 (1.82) | 119 |  |  |  |
|  | Int | Male | 4.886 (1.98) | 5.062 (1.8) | 181 |  |  |  |
|  | Cont | Male | 5.637 (1.93) | 5.522 (1.73) | 27 |  |  |  |
| Isolation | Int | Female | 6.249 (2.02) | 6.059 (1.9) | 276 | *p=0.82* | *p=0.464* | *p=0.008* |
|  | Cont | Female | 6.21 (2.13) | 6.147 (2.11) | 119 |  |  |  |
|  | Int | Male | 7.537 (1.93) | 6.924 (1.9) | 181 |  |  |  |
|  | Cont | Male | 6.238 (1.86) | 6.09 (1.97) | 27 |  |  |  |
| Mindfulness | Int | Female | 6.299 (1.75) | 5.93 (1.78) | 276 | *p=0.567* | *p=0.496* | *p=0.732* |
|  | Cont | Female | 6.399 (1.72) | 6.477 (1.85) | 119 |  |  |  |
|  | Int | Male | 5.745 (2.11) | 5.535 (1.96) | 181 |  |  |  |
|  | Cont | Male | 6.343 (1.99) | 6.104 (1.56) | 27 |  |  |  |
| Over Identification | Int | Female | 6.183 (1.58) | 5.863 (1.47) | 276 | *p=0.904* | *p=0.663* | *p=0.401* |
|  | Cont | Female | 6.255 (1.5) | 5.901 (1.65) | 119 |  |  |  |
|  | Int | Male | 6.476 (1.43) | 6.175 (1.39) | 181 |  |  |  |
|  | Cont | Male | 6.293 (1.18) | 5.949 (1.49) | 27 |  |  |  |
| Substance | Int | Female | 2.269 (0.82) | 2.935 (1.37) | 276 | *p=0.021* | *p=0.338* | *p=0.495* |
|  | Cont | Female | 2.362 (0.92) | 2.911 (1.14) | 119 |  |  |  |
|  | Int | Male | 2.526 (1.4) | 3.076 (1.31) | 181 |  |  |  |
|  | Cont | Male | 2.496 (1.16) | 3.337 (0.98) | 27 |  |  |  |

^1^Int = Intervention ^2^ Cont = Control

**Supplementary Table 5**

***Sensitivity Analysis for the effect of Age***

| Variable grouping | Condition | Age | Pre-treatment (SD) | Post-treatment (SD) | Sample (n) | Time*Condition (p-value) | Time*Age  (p-value) | Time*Condition*Age (p-value) |
| --- | --- | --- | --- | --- | --- | --- | --- | --- |
| IUS Total | Int^1^ | 12 | 26.216 (8.53) | 24.842 (9.03) | 37 | 0.591 | 0.596 | 0.166 |
|  | Cont^2^ | 12 | 20.5 (6.85) | 26.867 (7.57) | 2 |  |  |  |
|  | Int | 13 | 25.904 (8.45) | 25.382 (7.73) | 208 |  |  |  |
|  | Cont | 13 | 28.149 (9.38) | 29.352 (9.2) | 47 |  |  |  |
|  | Int | 14 | 30.264 (8.48) | 28.514 (9.06) | 72 |  |  |  |
|  | Cont | 14 | 27.049 (9.47) | 29.509 (10.13) | 61 |  |  |  |
|  | Int | 15 | 27.667 (7.04) | 24.039 (6.6) | 9 |  |  |  |
|  | Cont | 15 | 24.964 (9.45) | 27.876 (10.77) | 28 |  |  |  |
| Inhibitory IU | Int | 12 | 11.378 (4.3) | 10.71 (4.02) | 37 | **0.001** | 0.413 | 0.061 |
|  | Cont | 12 | 9.5 (3.69) | 9.656 (1.65) | 2 |  |  |  |
|  | Int | 13 | 10.75 (3.78) | 10.455 (3.45) | 208 |  |  |  |
|  | Cont | 13 | 11.957 (4.75) | 11.979 (4.32) | 47 |  |  |  |
|  | Int | 14 | 12.486 (3.69) | 11.74 (3.9) | 72 |  |  |  |
|  | Cont | 14 | 11.262 (4.46) | 12.337 (4.31) | 61 |  |  |  |
|  | Int | 15 | 12 (3.27) | 9.51 (2.49) | 9 |  |  |  |
|  | Cont | 15 | 9.714 (4.29) | 10.834 (4.24) | 28 |  |  |  |
| Prospective IU | Int | 12 | 14.838 (4.79) | 14.004 (5.62) | 37 | **0.001** | 0.572 | 0.653 |
|  | Cont | 12 | 11 (3.16) | 16.66 (2.58) | 2 |  |  |  |
|  | Int | 13 | 15.154 (5.17) | 14.776 (4.83) | 208 |  |  |  |
|  | Cont | 13 | 16.191 (5.38) | 17.168 (5.3) | 47 |  |  |  |
|  | Int | 14 | 17.778 (5.48) | 16.836 (5.55) | 72 |  |  |  |
|  | Cont | 14 | 15.787 (5.47) | 16.922 (6.06) | 61 |  |  |  |
|  | Int | 15 | 15.667 (4.24) | 14.122 (4.22) | 9 |  |  |  |
|  | Cont | 15 | 15.25 (5.81) | 16.275 (6.63) | 28 |  |  |  |
| CATS Social | Int | 12 | 17.892 (7.71) | 20.673 (9.61) | 37 | 0.118 | 0.051 | 0.195 |
|  | Cont | 12 | 16.645 (7.11) | 18.575 (6) | 2 |  |  |  |
|  | Int | 13 | 18.466 (8.7) | 20.718 (9.62) | 208 |  |  |  |
|  | Cont | 13 | 18.745 (8.6) | 20.912 (6.58) | 47 |  |  |  |
|  | Int | 14 | 21.708 (9.88) | 22.05 (9.26) | 72 |  |  |  |
|  | Cont | 14 | 21.279 (9.14) | 22.211 (9.34) | 61 |  |  |  |
|  | Int | 15 | 20.333 (11) | 15.639 (6.71) | 9 |  |  |  |
|  | Cont | 15 | 18.964 (8.71) | 20.008 (9.04) | 28 |  |  |  |
| CATS Personal | Int | 12 | 15.514 (7.58) | 21.888 (11.34) | 37 | 0.06 | 0.061 | 0.23 |
|  | Cont | 12 | 15.357 (8.44) | 22.623 (5.68) | 2 |  |  |  |
|  | Int | 13 | 16.875 (8.56) | 19.924 (9.37) | 208 |  |  |  |
|  | Cont | 13 | 16.723 (8.79) | 19.936 (6.36) | 47 |  |  |  |
|  | Int | 14 | 19.153 (8.54) | 19.897 (8.14) | 72 |  |  |  |
|  | Cont | 14 | 18.738 (8.68) | 21.97 (10.2) | 61 |  |  |  |
|  | Int | 15 | 16.889 (9.18) | 14.134 (4.88) | 9 |  |  |  |
|  | Cont | 15 | 19.321 (11.28) | 21.064 (10.31) | 28 |  |  |  |
| SCAS | Int | 12 | 26.919 (13.6) | 28.033 (16.23) | 37 | 0.389 | 0.234 | 0.091 |
|  | Cont | 12 | 24.5 (16.34) | 35.078 (17.11) | 2 |  |  |  |
|  | Int | 13 | 28.813 (16.48) | 27.068 (15.79) | 208 |  |  |  |
|  | Cont | 13 | 32.83 (14.52) | 34.928 (15.57) | 47 |  |  |  |
|  | Int | 14 | 34.375 (17.41) | 36.93 (18.75) | 72 |  |  |  |
|  | Cont | 14 | 33.475 (17.17) | 31.604 (16.19) | 61 |  |  |  |
|  | Int | 15 | 29 (15.28) | 22.486 (12.48) | 9 |  |  |  |
|  | Cont | 15 | 28.143 (20.62) | 27.657 (15.94) | 28 |  |  |  |
| Urgency | Int | 12 | 31.135 (6.81) | 30.873 (8.52) | 37 | 0.547 | 0.641 | 0.371 |
|  | Cont | 12 | 41 (5.27) | 28.913 (7.64) | 2 |  |  |  |
|  | Int | 13 | 30.611 (7.43) | 30.624 (7.64) | 208 |  |  |  |
|  | Cont | 13 | 27.809 (7.24) | 28.885 (6.36) | 47 |  |  |  |
|  | Int | 14 | 30.833 (6.39) | 31.874 (6.77) | 72 |  |  |  |
|  | Cont | 14 | 27.607 (6.65) | 27.372 (6.67) | 61 |  |  |  |
|  | Int | 15 | 28.222 (6.94) | 29.211 (6.24) | 9 |  |  |  |
|  | Cont | 15 | 26.357 (7.59) | 27.668 (7.36) | 28 |  |  |  |
| Envy | Int | 12 | 17.243 (6.9) | 17.93 (6.14) | 37 | 0.31 | 0.134 | 0.464 |
|  | Cont | 12 | 17.838 (9.72) | 19.727 (6.64) | 2 |  |  |  |
|  | Int | 13 | 17.572 (7.29) | 18.175 (6.99) | 208 |  |  |  |
|  | Cont | 13 | 18.319 (6.97) | 20.398 (6.72) | 47 |  |  |  |
|  | Int | 14 | 20.903 (7.09) | 20.815 (7.21) | 72 |  |  |  |
|  | Cont | 14 | 19.951 (7.81) | 19.682 (7.73) | 61 |  |  |  |
|  | Int | 15 | 17.556 (6.31) | 16.416 (5.3) | 9 |  |  |  |
|  | Cont | 15 | 17.643 (7.79) | 18.544 (8.13) | 28 |  |  |  |
| GAD SCAS | Int | 12 | 5.703 (3.46) | 6.474 (3.87) | 37 | 0.364 | 0.167 | 0.026 |
|  | Cont | 12 | 4.5 (3.69) | 6.652 (3.19) | 2 |  |  |  |
|  | Int | 13 | 6.635 (3.62) | 6.014 (3.29) | 208 |  |  |  |
|  | Cont | 13 | 6.851 (3.36) | 7.235 (3.25) | 47 |  |  |  |
|  | Int | 14 | 7.792 (3.69) | 8.462 (4.09) | 72 |  |  |  |
|  | Cont | 14 | 7.934 (4.13) | 7.144 (3.64) | 61 |  |  |  |
|  | Int | 15 | 6.889 (2.88) | 5.317 (2.26) | 9 |  |  |  |
|  | Cont | 15 | 7.179 (4.11) | 6.255 (3.24) | 28 |  |  |  |
| OCD SCAS | Int | 12 | 4.676 (2.69) | 4.669 (3.48) | 37 | 0.381 | 0.406 | 0.158 |
|  | Cont | 12 | 4 (4.22) | 2.355 (2.08) | 2 |  |  |  |
|  | Int | 13 | 5 (3.53) | 4.742 (3.14) | 208 |  |  |  |
|  | Cont | 13 | 5.745 (4.04) | 5.287 (3.33) | 47 |  |  |  |
|  | Int | 14 | 5.847 (3.84) | 5.764 (3.99) | 72 |  |  |  |
|  | Cont | 14 | 5.475 (3.68) | 4.92 (3.08) | 61 |  |  |  |
|  | Int | 15 | 5.222 (2.81) | 3.334 (1.93) | 9 |  |  |  |
|  | Cont | 15 | 4.429 (4.04) | 4.437 (3.01) | 28 |  |  |  |
| SA SCAS | Int | 12 | 6.405 (3.47) | 6.998 (3.96) | 37 | 0.599 | 0.406 | 0.328 |
|  | Cont | 12 | 7.5 (2.64) | 7.017 (3.66) | 2 |  |  |  |
|  | Int | 13 | 7.048 (3.75) | 6.932 (3.65) | 208 |  |  |  |
|  | Cont | 13 | 8.489 (3.25) | 7.726 (3.52) | 47 |  |  |  |
|  | Int | 14 | 7.944 (3.6) | 8.334 (3.51) | 72 |  |  |  |
|  | Cont | 14 | 8.197 (3.67) | 7.836 (3.62) | 61 |  |  |  |
|  | Int | 15 | 6 (3.78) | 4.848 (2.92) | 9 |  |  |  |
|  | Cont | 15 | 6.893 (3.82) | 6.682 (3.31) | 28 |  |  |  |
| AFQ | Int | 12 | 32.811 (11.96) | 36.138 (13.2) | 37 | 0.062 | 0.424 | 0.527 |
|  | Cont | 12 | 23.381 (8.69) | 40.131 (9.98) | 2 |  |  |  |
|  | Int | 13 | 35.379 (12.5) | 35.917 (13.59) | 208 |  |  |  |
|  | Cont | 13 | 37.383 (12.17) | 39.564 (11.56) | 47 |  |  |  |
|  | Int | 14 | 39.806 (12.73) | 41.264 (13.13) | 72 |  |  |  |
|  | Cont | 14 | 38.902 (11.63) | 43.102 (12.96) | 61 |  |  |  |
|  | Int | 15 | 36.444 (11.34) | 34.091 (10.35) | 9 |  |  |  |
|  | Cont | 15 | 36.536 (15.56) | 39.659 (11.39) | 28 |  |  |  |
| DDI | Int | 12 | 41.946 (12.31) | 43.388 (9.23) | 37 | 0.578 | 0.54 | 0.36 |
|  | Cont | 12 | 41.988 (8.21) | 42.128 (10.4) | 2 |  |  |  |
|  | Int | 13 | 42.719 (11.61) | 41.373 (11.21) | 208 |  |  |  |
|  | Cont | 13 | 42.128 (13.66) | 44.009 (10.77) | 47 |  |  |  |
|  | Int | 14 | 40.75 (12.49) | 40.787 (12.86) | 72 |  |  |  |
|  | Cont | 14 | 42.493 (12.12) | 41.493 (11.83) | 61 |  |  |  |
|  | Int | 15 | 50.444 (10.52) | 48.473 (9.07) | 9 |  |  |  |
|  | Cont | 15 | 39.393 (9.76) | 38.191 (10.89) | 28 |  |  |  |
| RTQ | Int | 12 | 21.753 (9.61) | 23.83 (8.61) | 37 | 0.011 | 0.303 | 0.128 |
|  | Cont | 12 | 20.283 (11.3) | 24.744 (6.09) | 2 |  |  |  |
|  | Int | 13 | 23.522 (9.48) | 25.174 (9.13) | 208 |  |  |  |
|  | Cont | 13 | 24.468 (9.78) | 27.909 (10.08) | 47 |  |  |  |
|  | Int | 14 | 26.833 (8.96) | 27.524 (9.66) | 72 |  |  |  |
|  | Cont | 14 | 26.15 (10.49) | 28.727 (8.63) | 61 |  |  |  |
|  | Int | 15 | 26.222 (8.97) | 22.451 (7.85) | 9 |  |  |  |
|  | Cont | 15 | 25.5 (10.16) | 29.58 (8.61) | 28 |  |  |  |
| BFNE | Int | 12 | 16.595 (8.18) | 19.366 (8.1) | 37 | 0.135 | 0.059 | 0.193 |
|  | Cont | 12 | 13.632 (7.74) | 22.996 (4.66) | 2 |  |  |  |
|  | Int | 13 | 17.652 (7.96) | 20.032 (8.27) | 208 |  |  |  |
|  | Cont | 13 | 18.298 (7.98) | 20.611 (7.42) | 47 |  |  |  |
|  | Int | 14 | 21.403 (8.15) | 22.07 (7.83) | 72 |  |  |  |
|  | Cont | 14 | 21.802 (8.47) | 21.925 (6.74) | 61 |  |  |  |
|  | Int | 15 | 20.889 (10.77) | 18.599 (7.25) | 9 |  |  |  |
|  | Cont | 15 | 18.357 (8.4) | 21.185 (5.86) | 28 |  |  |  |
| Pos coping Anger | Int | 12 | 16.919 (4.61) | 17.735 (4.89) | 37 | 0.128 | 0.367 | 0.06 |
|  | Cont | 12 | 16.335 (2.78) | 17.3 (2.19) | 2 |  |  |  |
|  | Int | 13 | 17.603 (4.41) | 17.773 (4.49) | 208 |  |  |  |
|  | Cont | 13 | 18.894 (3.78) | 18.576 (3.92) | 47 |  |  |  |
|  | Int | 14 | 18.569 (3.26) | 18.072 (3.41) | 72 |  |  |  |
|  | Cont | 14 | 17.715 (4.11) | 17.685 (4.38) | 61 |  |  |  |
|  | Int | 15 | 19.889 (2.1) | 17.196 (4.04) | 9 |  |  |  |
|  | Cont | 15 | 16.107 (4.43) | 16.609 (3.48) | 28 |  |  |  |
| SCS | Int | 12 | 3.207 (0.51) | 3.018 (0.58) | 37 | 0.559 | 0.618 | 0.514 |
|  | Int | 12 | 3.212 (0.44) | 3.082 (0.72) | 2 |  |  |  |
|  | Cont | 13 | 3.179 (0.6) | 3.053 (0.55) | 208 |  |  |  |
|  | Int | 13 | 3.261 (0.59) | 3.174 (0.63) | 47 |  |  |  |
|  | Cont | 14 | 3.023 (0.51) | 2.882 (0.54) | 72 |  |  |  |
|  | Int | 14 | 3.037 (0.58) | 2.967 (0.66) | 61 |  |  |  |
|  | Cont | 15 | 3.185 (0.58) | 3.244 (0.31) | 9 |  |  |  |
|  | Int | 15 | 3.012 (0.68) | 2.893 (0.66) | 28 |  |  |  |
| SMFQ | Cont | 12 | 4.676 (5.11) | 7.355 (6.96) | 37 | 0.117 | 0.271 | 0.195 |
|  | Int | 12 | 6.282 (6.46) | 8.152 (5.25) | 2 |  |  |  |
|  | Cont | 13 | 5.546 (5.42) | 8.058 (6.1) | 208 |  |  |  |
|  | Int | 13 | 5.021 (4.92) | 7.064 (6.13) | 47 |  |  |  |
|  | Cont | 14 | 8.292 (5.72) | 9.383 (6) | 72 |  |  |  |
|  | Int | 14 | 7.551 (5.92) | 9.396 (5.83) | 61 |  |  |  |
|  | Cont | 15 | 6.111 (6.66) | 5.252 (4.58) | 9 |  |  |  |
|  | Int | 15 | 6.714 (5.99) | 9.122 (5.28) | 28 |  |  |  |
| Public Sharing | Cont | 12 | 5.555 (2.22) | 6.63 (2.76) | 37 | 0.033 | 0.084 | 0.059 |
|  | Int | 12 | 5.862 (2.4) | 6.376 (2.48) | 2 |  |  |  |
|  | Cont | 13 | 6.376 (2.47) | 6.293 (2.31) | 208 |  |  |  |
|  | Int | 13 | 7.255 (2.11) | 7.242 (1.86) | 47 |  |  |  |
|  | Cont | 14 | 7.369 (1.8) | 6.752 (2.21) | 72 |  |  |  |
|  | Int | 14 | 6.77 (1.85) | 6.376 (2.24) | 61 |  |  |  |
|  | Cont | 15 | 7.222 (2.22) | 5.936 (2.21) | 9 |  |  |  |
|  | Int | 15 | 5.571 (1.5) | 6.303 (2.14) | 28 |  |  |  |
| Surveillance SM | Cont | 12 | 10.189 (4.42) | 13.114 (6.02) | 37 | 0.351 | 0.113 | 0.298 |
|  | Int | 12 | 12.023 (3.16) | 13.12 (4.65) | 2 |  |  |  |
|  | Cont | 13 | 10.705 (4.61) | 11.688 (5.13) | 208 |  |  |  |
|  | Int | 13 | 12.307 (3.55) | 12.351 (3.81) | 47 |  |  |  |
|  | Cont | 14 | 13.047 (3.65) | 12.874 (4.38) | 72 |  |  |  |
|  | Int | 14 | 12.131 (4.37) | 11.569 (4.64) | 61 |  |  |  |
|  | Cont | 15 | 12.556 (3.54) | 11.649 (5) | 9 |  |  |  |
|  | Int | 15 | 11.071 (3.36) | 11.377 (3.67) | 28 |  |  |  |
| Upset share online | Cont | 12 | 1.324 (0.84) | 1.723 (0.94) | 37 | **0.001** | 0.079 | **0** |
|  | Int | 12 | 0.971 (0.2) | 1.764 (0.87) | 2 |  |  |  |
|  | Cont | 13 | 1.434 (0.81) | 1.68 (0.99) | 208 |  |  |  |
|  | Int | 13 | 2.319 (1.06) | 1.455 (0.72) | 47 |  |  |  |
|  | Cont | 14 | 2.639 (1.05) | 1.607 (1) | 72 |  |  |  |
|  | Int | 14 | 1.721 (0.83) | 1.606 (0.87) | 61 |  |  |  |
|  | Cont | 15 | 2.778 (0.93) | 1.545 (0.8) | 9 |  |  |  |
|  | Int | 15 | 1.179 (0.47) | 1.694 (0.85) | 28 |  |  |  |
| Textpriv | Cont | 12 | 2.054 (1.25) | 2.746 (1.53) | 37 | 0.038 | 0.425 | 0.005 |
|  | Int | 12 | 2.65 (1.27) | 3.653 (1.9) | 2 |  |  |  |
|  | Cont | 13 | 2.235 (1.12) | 2.686 (1.57) | 208 |  |  |  |
|  | Int | 13 | 1.626 (1) | 2.569 (1.1) | 47 |  |  |  |
|  | Cont | 14 | 1.722 (0.8) | 2.874 (1.26) | 72 |  |  |  |
|  | Int | 14 | 2.164 (1.12) | 2.754 (1.11) | 61 |  |  |  |
|  | Cont | 15 | 1.333 (0.67) | 2.551 (1.1) | 9 |  |  |  |
|  | Int | 15 | 2.607 (0.86) | 2.732 (0.89) | 28 |  |  |  |
| SM Reduction | Cont | 12 | 1.892 (0.96) | 2.092 (1.09) | 37 | 0.284 | 0.5 | 0.305 |
|  | Int | 12 | 1.721 (0.9) | 2.161 (1.14) | 2 |  |  |  |
|  | Cont | 13 | 2.048 (1.1) | 2.003 (1.09) | 208 |  |  |  |
|  | Int | 13 | 1.872 (1.09) | 1.792 (0.95) | 47 |  |  |  |
|  | Cont | 14 | 2.25 (1.13) | 2.122 (1.12) | 72 |  |  |  |
|  | Int | 14 | 2.016 (1.21) | 2.254 (1.08) | 61 |  |  |  |
|  | Cont | 15 | 2.111 (1.01) | 1.908 (1.13) | 9 |  |  |  |
|  | Int | 15 | 1.929 (1.14) | 1.993 (1.11) | 28 |  |  |  |
| FOMO | Cont | 12 | 3.324 (1.44) | 4.685 (2.24) | 37 | 0.304 | 0.345 | 0.02 |
|  | Int | 12 | 4.01 (0.84) | 3.834 (1.31) | 2 |  |  |  |
|  | Cont | 13 | 3.968 (1.98) | 4.391 (2.09) | 208 |  |  |  |
|  | Int | 13 | 5.17 (1.88) | 5.195 (2.13) | 47 |  |  |  |
|  | Cont | 14 | 5.097 (1.85) | 5.129 (1.83) | 72 |  |  |  |
|  | Int | 14 | 4.803 (1.92) | 4.859 (2.24) | 61 |  |  |  |
|  | Cont | 15 | 5.333 (1.65) | 4.654 (2.09) | 9 |  |  |  |
|  | Int | 15 | 3.857 (1.87) | 4.47 (2.02) | 28 |  |  |  |
| Neg_eff_SM | Cont | 12 | 2.027 (2.08) | 3.21 (2.63) | 37 | 0.202 | 0.342 | 0.347 |
|  | Int | 12 | 2.789 (1.19) | 5.985 (2.83) | 2 |  |  |  |
|  | Cont | 13 | 2.602 (2.7) | 3.53 (2.64) | 208 |  |  |  |
|  | Int | 13 | 3.213 (2.6) | 2.912 (2.55) | 47 |  |  |  |
|  | Cont | 14 | 3.708 (3.14) | 3.766 (3.01) | 72 |  |  |  |
|  | Int | 14 | 3.607 (3.03) | 3.429 (2.75) | 61 |  |  |  |
|  | Cont | 15 | 2.444 (2.39) | 2.784 (2.82) | 9 |  |  |  |
|  | Int | 15 | 2.357 (2.23) | 2.495 (2.29) | 28 |  |  |  |
| Mean acts online | Cont | 12 | 4.541 (2.31) | 4.74 (2.09) | 37 | 0.628 | 0.506 | 0.553 |
|  | Int | 12 | 3.96 (1.29) | 4.68 (1.76) | 2 |  |  |  |
|  | Cont | 13 | 4.385 (2.14) | 4.959 (2.04) | 208 |  |  |  |
|  | Int | 13 | 4.553 (2.76) | 4.81 (2.37) | 47 |  |  |  |
|  | Cont | 14 | 4.431 (2.56) | 5.28 (2.7) | 72 |  |  |  |
|  | Int | 14 | 4.213 (1.95) | 4.683 (2.1) | 61 |  |  |  |
|  | Cont | 15 | 3.778 (1.04) | 4.226 (2.09) | 9 |  |  |  |
|  | Int | 15 | 3.75 (1.48) | 4.734 (2.59) | 28 |  |  |  |
| SK | Cont | 12 | 5.589 (1.96) | 5.083 (1.78) | 37 | 0.346 | 0.609 | 0.475 |
|  | Int | 12 | 7.651 (1.4) | 5.356 (1.28) | 2 |  |  |  |
|  | Cont | 13 | 5.623 (1.88) | 5.301 (1.84) | 208 |  |  |  |
|  | Int | 13 | 6.128 (1.61) | 5.854 (1.72) | 47 |  |  |  |
|  | Cont | 14 | 5.939 (1.52) | 5.511 (1.47) | 72 |  |  |  |
|  | Int | 14 | 6.24 (1.47) | 5.785 (1.79) | 61 |  |  |  |
|  | Cont | 15 | 7.556 (1.18) | 6.401 (2.33) | 9 |  |  |  |
|  | Int | 15 | 5.714 (1.47) | 5.492 (1.62) | 28 |  |  |  |
| SJ | Cont | 12 | 7.629 (2.05) | 6.674 (1.95) | 37 | 0.152 | 0.251 | **0.001** |
|  | Int | 12 | 5.984 (1.4) | 6.037 (1.79) | 2 |  |  |  |
|  | Cont | 13 | 7.248 (2.19) | 6.64 (2.02) | 208 |  |  |  |
|  | Int | 13 | 6.894 (2.22) | 6.913 (2.42) | 47 |  |  |  |
|  | Cont | 14 | 6.168 (1.97) | 6.18 (2.03) | 72 |  |  |  |
|  | Int | 14 | 6.161 (1.99) | 6.253 (2.17) | 61 |  |  |  |
|  | Cont | 15 | 5.556 (1.85) | 6.613 (1.71) | 9 |  |  |  |
|  | Int | 15 | 6.25 (2.68) | 5.394 (1.97) | 28 |  |  |  |
| Common Humanity | Cont | 12 | 4.488 (2.09) | 5.14 (1.76) | 37 |  |  |  |
|  | Int | 12 | 6.37 (1.16) | 5.444 (1.82) | 2 | 0.322 | 0.096 | 0.24 |
|  | Cont | 13 | 5.007 (1.87) | 5.327 (1.79) | 208 |  |  |  |
|  | Int | 13 | 5.872 (2.1) | 5.95 (2.08) | 47 |  |  |  |
|  | Cont | 14 | 5.856 (1.7) | 5.506 (1.52) | 72 |  |  |  |
|  | Int | 14 | 5.889 (1.52) | 5.541 (1.61) | 61 |  |  |  |
|  | Cont | 15 | 7.111 (1.21) | 6.248 (2.32) | 9 |  |  |  |
|  | Int | 15 | 5.393 (1.57) | 5.813 (1.75) | 28 |  |  |  |
| Isolation | Cont | 12 | 7.573 (2.02) | 6.745 (1.97) | 37 | 0.427 | 0.221 | 0.097 |
|  | Int | 12 | 7.143 (1.52) | 6.377 (1.15) | 2 |  |  |  |
|  | Cont | 13 | 7.04 (2.02) | 6.586 (1.92) | 208 |  |  |  |
|  | Int | 13 | 6.745 (2.03) | 6.594 (2.19) | 47 |  |  |  |
|  | Cont | 14 | 5.852 (1.88) | 5.767 (1.96) | 72 |  |  |  |
|  | Int | 14 | 5.708 (2.05) | 5.946 (1.98) | 61 |  |  |  |
|  | Cont | 15 | 6.444 (1.85) | 6.899 (1.69) | 9 |  |  |  |
|  | Int | 15 | 6.393 (2.03) | 5.596 (1.98) | 28 |  |  |  |
| Mindfulness | Cont | 12 | 5.664 (2.15) | 5.501 (1.82) | 37 | 0.155 | 0.278 | 0.279 |
|  | Int | 12 | 6.724 (1.04) | 6.419 (2.07) | 2 |  |  |  |
|  | Cont | 13 | 5.966 (1.96) | 5.912 (1.94) | 208 |  |  |  |
|  | Int | 13 | 6.638 (2.05) | 6.551 (2.04) | 47 |  |  |  |
|  | Cont | 14 | 6.373 (1.75) | 5.564 (1.77) | 72 |  |  |  |
|  | Int | 14 | 6.348 (1.49) | 6.315 (1.76) | 61 |  |  |  |
|  | Cont | 15 | 6.222 (1.49) | 5.934 (2.14) | 9 |  |  |  |
|  | Int | 15 | 5.964 (1.89) | 6.356 (1.62) | 28 |  |  |  |
| Overidentification | Cont | 12 | 6.708 (1.28) | 6.004 (1.27) | 37 |  |  |  |
|  | Int | 12 | 6.79 (1.79) | 5.311 (1.28) | 2 | 0.47 | 0.595 | 0.64 |
|  | Cont | 13 | 6.42 (1.55) | 6.078 (1.44) | 208 |  |  |  |
|  | Int | 13 | 6.489 (1.47) | 6.131 (1.47) | 47 |  |  |  |
|  | Cont | 14 | 5.972 (1.32) | 5.754 (1.44) | 72 |  |  |  |
|  | Int | 14 | 6.175 (1.39) | 5.848 (1.7) | 61 |  |  |  |
|  | Cont | 15 | 6.556 (1.59) | 6.451 (1.08) | 9 |  |  |  |
|  | Int | 15 | 5.929 (1.49) | 5.496 (1.56) | 28 |  |  |  |
| Substance | Cont | 12 | 2.407 (1.01) | 2.882 (1.15) | 37 | 0.4 | 0.227 | 0.382 |
|  | Int | 12 | 2.519 (0.72) | 3.282 (0.92) | 2 |  |  |  |
|  | Cont | 13 | 2.474 (1.34) | 2.973 (1.26) | 208 |  |  |  |
|  | Int | 13 | 2.207 (0.71) | 2.562 (1.04) | 47 |  |  |  |
|  | Cont | 14 | 2.444 (1.03) | 3.055 (1.59) | 72 |  |  |  |
|  | Int | 14 | 2.488 (1.04) | 3.11 (1.11) | 61 |  |  |  |
|  | Cont | 15 | 2.444 (0.97) | 3.026 (1.94) | 9 |  |  |  |
|  |  | 15 | 2.393 (1.08) | 3.514 (1.06) | 28 |  |  |  |

^1^Int = Intervention ^2^ Cont = Control

| **Supplementary Table 6:**  *Contrasting Effects Associated With Intervention Fidelity* | | | | | | | | |
| --- | --- | --- | --- | --- | --- | --- | --- | --- |
|  |  |  |  |  | Contrasts |  |  |  |
| Outcome variable | Condition (levels of fidelity) | Pre-treatment (SD) | Post-treatment (SD) | Sample (n) | "Less than Half" of lessons vs control | "Half or more" of lessons vs control | "Completed all lessons" vs Control | |
| IUS_Total | Control | 26.726 (9.35) | 28.979 (9.74) | 146 | 0.423 | **0.004** | **<.001** | |
|  | LessThanHalf | 27.643 (8.2) | 28.071 (10.46) | 14 |  |  |  | |
|  | HalfOrMore | 26.983 (7.61) | 25.796 (7.89) | 60 |  |  |  | |
|  | Complete | 30.607 (8.04) | 28.094 (8.36) | 56 |  |  |  | |
| IUS_Inhibitory | Control | 11.062 (4.54) | 11.878 (4.22) | 146 | 0.697 | **0.002** | **0.001** | |
|  | LessThanHalf | 11.643 (3.7) | 12.143 (4.78) | 14 |  |  |  | |
|  | HalfOrMore | 11.283 (3.64) | 10.498 (3.45) | 60 |  |  |  | |
|  | Complete | 12.661 (3.61) | 11.402 (3.57) | 56 |  |  |  | |
| IUS_Prospective | Control | 15.664 (5.41) | 16.761 (5.81) | 146 | 0.34 | 0.026 | **0.002** | |
|  | LessThanHalf | 16 (5.02) | 15.929 (6.41) | 14 |  |  |  | |
|  | HalfOrMore | 15.7 (4.53) | 15.161 (5.05) | 60 |  |  |  | |
|  | Complete | 17.946 (5.22) | 16.644 (5.22) | 56 |  |  |  | |
| CATS_Social | Control | 19.906 (8.88) | 21.157 (8.4) | 146 | 0.851 | 0.351 | 0.203 | |
|  | LessThanHalf | 18.211 (8) | 19.229 (8.7) | 14 |  |  |  | |
|  | HalfOrMore | 21.117 (8.68) | 21.688 (9.64) | 60 |  |  |  | |
|  | Complete | 22.196 (9.95) | 21.073 (10.17) | 56 |  |  |  | |
| CATS_Personal | Control | 18.135 (9.2) | 21.057 (8.98) | 146 | 0.407 | 0.26 | 0.096 | |
|  | LessThanHalf | 17.217 (6.69) | 16.7 (7.63) | 14 |  |  |  | |
|  | HalfOrMore | 19.717 (9.75) | 20.714 (9.74) | 60 |  |  |  | |
|  | Complete | 19.071 (8.42) | 18.631 (8.29) | 56 |  |  |  | |
| SCAS_TOTA | Control | 31.753 (16.98) | 31.845 (15.85) | 146 | 0.062 | 0.027 | 0.192 | |
|  | LessThanHalf | 29.571 (14.28) | 25.357 (15.16) | 14 |  |  |  | |
|  | HalfOrMore | 33.333 (18.32) | 30.149 (14.97) | 60 |  |  |  | |
|  | Complete | 33.375 (16.45) | 36.764 (20.75) | 56 |  |  |  | |
| UPPS_Urgency | Control | 27.548 (7.1) | 27.899 (6.71) | 146 | 0.795 | 0.657 | 0.512 | |
|  | LessThanHalf | 33.571 (5.57) | 33.929 (5.51) | 14 |  |  |  | |
|  | HalfOrMore | 33.183 (6.3) | 32.373 (7.02) | 60 |  |  |  | |
|  | Complete | 30.304 (6.11) | 30.867 (6.67) | 56 |  |  |  | |
| DES_Total | Control | 18.895 (7.53) | 19.643 (7.44) | 146 | 0.22 | 0.261 | 0.09 | |
|  | LessThanHalf | 15.714 (5.43) | 19.214 (7.79) | 14 |  |  |  | |
|  | HalfOrMore | 20.117 (7.01) | 19.612 (7.62) | 60 |  |  |  | |
|  | Complete | 20.804 (6.91) | 19.851 (6.82) | 56 |  |  |  | |
| SCAS_GAD_ | Control | 7.342 (3.83) | 6.966 (3.41) | 146 | 0.195 | 0.609 | 0.057 | |
|  | LessThanHalf | 7.5 (3.71) | 6.071 (3.22) | 14 |  |  |  | |
|  | HalfOrMore | 7.317 (3.4) | 6.771 (3.47) | 60 |  |  |  | |
|  | Complete | 7.875 (3.46) | 8.539 (4.36) | 56 |  |  |  | |
| SCAS_OCD_ | Control | 5.253 (3.89) | 4.917 (3.13) | 146 | 0.841 | 0.131 | 0.223 | |
|  | LessThanHalf | 5.143 (3.75) | 4.929 (3.33) | 14 |  |  |  | |
|  | HalfOrMore | 5.967 (4.1) | 5.183 (3.3) | 60 |  |  |  | |
|  | Complete | 5.321 (3.16) | 5.463 (4.24) | 56 |  |  |  | |
| SCAS_SA_m | Control | 7.938 (3.58) | 7.512 (3.54) | 146 | 0.594 | 0.919 | 0.21 | |
|  | LessThanHalf | 6.786 (3.32) | 5.929 (3.37) | 14 |  |  |  | |
|  | HalfOrMore | 7.967 (4.02) | 7.593 (3.42) | 60 |  |  |  | |
|  | Complete | 7.482 (3.4) | 7.747 (3.85) | 56 |  |  |  | |
| AFQ_Total | Control | 37.594 (12.66) | 40.933 (12.11) | 146 | 0.744 | 0.289 | 0.468 | |
|  | LessThanHalf | 36.136 (12.46) | 37.411 (14.03) | 14 |  |  |  | |
|  | HalfOrMore | 37.917 (14.3) | 37.83 (13.53) | 60 |  |  |  | |
|  | Complete | 40.268 (11.24) | 41.41 (13.29) | 56 |  |  |  | |
| DDI_Total | Control | 42.048 (12.23) | 42.17 (11.58) | 146 | 0.24 | 0.531 | 0.865 | |
|  | LessThanHalf | 44.463 (15.43) | 48.071 (11.79) | 14 |  |  |  | |
|  | HalfOrMore | 41.517 (13.68) | 41.889 (12.05) | 60 |  |  |  | |
|  | Complete | 40.5 (12.1) | 41.057 (12.26) | 56 |  |  |  | |
| RTQ_Total | Control | 25.238 (10.16) | 28.442 (9.12) | 146 | 0.576 | 0.657 | 0.239 | |
|  | LessThanHalf | 24.675 (8.4) | 24.857 (10.04) | 14 |  |  |  | |
|  | HalfOrMore | 25.3 (10.59) | 26.685 (9.58) | 60 |  |  |  | |
|  | Complete | 26.446 (8.27) | 26.664 (9.4) | 56 |  |  |  | |
| BFNE_Total | Control | 19.707 (8.46) | 21.2 (6.85) | 146 | 0.038 | 0.871 | 0.036 | |
|  | LessThanHalf | 17.255 (6.16) | 22.143 (7.94) | 14 |  |  |  | |
|  | HalfOrMore | 20.667 (8.06) | 22.334 (7.7) | 60 |  |  |  | |
|  | Complete | 21.893 (8.17) | 21.038 (7.92) | 56 |  |  |  | |
| Positive coping Anger_Tot | Control | 17.885 (4.1) | 17.864 (4.11) | 146 | **0.002** | 0.3 | 0.186 | |
|  | LessThanHalf | 16.749 (2.65) | 19.429 (3.38) | 14 |  |  |  | |
|  | HalfOrMore | 17.083 (4.34) | 17.546 (3.72) | 60 |  |  |  | |
|  | Complete | 19.214 (3.11) | 18.342 (3.47) | 56 |  |  |  | |
| SCSTotal_ | Control | 3.126 (0.62) | 3.041 (0.66) | 146 | 0.522 | 0.897 | 0.979 | |
|  | LessThanHalf | 3.221 (0.5) | 3.083 (0.66) | 14 |  |  |  | |
|  | HalfOrMore | 2.956 (0.54) | 2.889 (0.51) | 60 |  |  |  | |
|  | Complete | 3.118 (0.52) | 3.004 (0.5) | 56 |  |  |  | |
| SMFQ_Tota | Control | 6.583 (5.7) | 8.4 (5.88) | 146 | 0.913 | 0.81 | 0.861 | |
|  | LessThanHalf | 6.363 (6.52) | 6.429 (5.1) | 14 |  |  |  | |
|  | HalfOrMore | 7.65 (6.13) | 8.681 (6.51) | 60 |  |  |  | |
|  | Complete | 7.75 (5.71) | 8.482 (6.21) | 56 |  |  |  | |
| Public Sharing | Control | 6.649 (1.99) | 6.584 (2.14) | 146 | 0.381 | 0.098 | 0.111 | |
|  | LessThanHalf | 6.748 (2.06) | 7.313 (2.79) | 14 |  |  |  | |
|  | HalfOrMore | 7.333 (2.62) | 6.543 (2.2) | 60 |  |  |  | |
|  | Complete | 7.563 (1.65) | 6.907 (2.13) | 56 |  |  |  | |
| Surveillance SM | Control | 11.976 (3.98) | 11.693 (4.28) | 146 | 0.036 | 0.203 | 0.628 | |
|  | LessThanHalf | 12.318 (4.32) | 13.938 (4.97) | 14 |  |  |  | |
|  | HalfOrMore | 12.4 (4.46) | 12.507 (5.48) | 60 |  |  |  | |
|  | Complete | 13.589 (3.31) | 13.041 (4.29) | 56 |  |  |  | |
| Upset share online | Control | 1.842 (0.99) | 1.551 (0.81) | 146 | 0.966 | 0.356 | **0** | |
|  | LessThanHalf | 1.615 (1.01) | 1.251 (0.6) | 14 |  |  |  | |
|  | HalfOrMore | 2.067 (1.17) | 1.606 (0.92) | 60 |  |  |  | |
|  | Complete | 2.964 (0.91) | 1.62 (0.99) | 56 |  |  |  | |
| Text private_ | Control | 2.073 (1.1) | 2.711 (1.1) | 146 | 0.42 | 0.987 | 0.002 | |
|  | LessThanHalf | 2.231 (1.18) | 3.227 (1.85) | 14 |  |  |  | |
|  | HalfOrMore | 2.2 (1.21) | 2.823 (1.65) | 60 |  |  |  | |
|  | Complete | 1.661 (0.74) | 2.904 (1.18) | 56 |  |  |  | |
| SM Reduction | Control | 1.941 (1.14) | 2.025 (1.06) | 146 | 0.605 | 0.664 | 0.46 | |
|  | LessThanHalf | 1.571 (0.83) | 1.429 (0.91) | 14 |  |  |  | |
|  | HalfOrMore | 1.883 (1.02) | 2.06 (1.16) | 60 |  |  |  | |
|  | Complete | 2.179 (1.17) | 2.063 (1.01) | 56 |  |  |  | |
| FOMO_modi | Control | 4.699 (1.96) | 4.828 (2.2) | 146 | 0.672 | 0.248 | 0.148 | |
|  | LessThanHalf | 4.324 (1.76) | 4.625 (2.03) | 14 |  |  |  | |
|  | HalfOrMore | 4.4 (2.19) | 4.946 (2.28) | 60 |  |  |  | |
|  | Complete | 5.536 (1.7) | 5.269 (1.83) | 56 |  |  |  | |
| Neg_eff_SM | Control | 3.12 (2.82) | 3.062 (2.62) | 146 | 0.217 | 0.23 | 0.214 | |
|  | LessThanHalf | 2.041 (2) | 2.585 (2.61) | 14 |  |  |  | |
|  | HalfOrMore | 3.133 (3.29) | 3.704 (2.9) | 60 |  |  |  | |
|  | Complete | 3.429 (2.9) | 3.8 (3.03) | 56 |  |  |  | |
| Mean Acts Online | Control | 4.212 (2.15) | 4.752 (2.3) | 146 | 0.634 | 0.769 | 0.176 | |
|  | LessThanHalf | 3.5 (1.56) | 4.071 (1.92) | 14 |  |  |  | |
|  | HalfOrMore | 4.417 (2.2) | 4.96 (2.51) | 60 |  |  |  | |
|  | Complete | 4.411 (2.57) | 5.152 (2.6) | 56 |  |  |  | |
| SCS_SK_mo | Control | 6.164 (1.55) | 5.797 (1.73) | 146 | 0.335 | 0.637 | 0.313 | |
|  | LessThanHalf | 5.223 (1.1) | 5.5 (1.97) | 14 |  |  |  | |
|  | HalfOrMore | 5.433 (1.73) | 5.137 (1.62) | 60 |  |  |  | |
|  | Complete | 6.446 (1.48) | 5.818 (1.64) | 56 |  |  |  | |
| SCS_SJ_mo | Control | 6.478 (2.23) | 6.324 (2.29) | 146 | 0.574 | 0.497 | 0.302 | |
|  | LessThanHalf | 7.413 (1.93) | 7.143 (2.22) | 14 |  |  |  | |
|  | HalfOrMore | 6.467 (2.35) | 6.151 (1.89) | 60 |  |  |  | |
|  | Complete | 6.125 (1.91) | 6.281 (2) | 56 |  |  |  | |
| Common Humanity_mo | Control | 5.829 (1.76) | 5.732 (1.8) | 146 | 0.711 | 0.456 | 0.152 | |
|  | LessThanHalf | 5.071 (1.45) | 5.143 (1.78) | 14 |  |  |  | |
|  | HalfOrMore | 5.167 (2.03) | 5.367 (1.7) | 60 |  |  |  | |
|  | Complete | 6.304 (1.74) | 5.792 (1.67) | 56 |  |  |  | |
| Isolation_m | Control | 6.215 (2.08) | 6.136 (2.09) | 146 | 0.117 | 0.849 | 0.251 | |
|  | LessThanHalf | 7.195 (1.97) | 5.857 (2.05) | 14 |  |  |  | |
|  | HalfOrMore | 6.45 (2.15) | 6.238 (2.01) | 60 |  |  |  | |
|  | Complete | 5.893 (1.82) | 6.088 (1.9) | 56 |  |  |  | |
| Mindfulness_ | Control | 6.388 (1.77) | 6.408 (1.81) | 146 | 0.769 | 0.199 | 0.008 | |
|  | LessThanHalf | 6.76 (1.42) | 6.643 (1.5) | 14 |  |  |  | |
|  | HalfOrMore | 5.667 (1.88) | 5.454 (1.73) | 60 |  |  |  | |
|  | Complete | 6.625 (1.51) | 5.796 (1.86) | 56 |  |  |  | |
| Overidentification_mo | Control | 6.262 (1.44) | 5.91 (1.62) | 146 | 0.572 | 0.144 | 0.617 | |
|  | LessThanHalf | 6.035 (1.46) | 5.929 (1.84) | 14 |  |  |  | |
|  | HalfOrMore | 5.717 (1.42) | 5.815 (1.27) | 60 |  |  |  | |
|  | Complete | 6.286 (1.35) | 5.861 (1.36) | 56 |  |  |  | |
| Substance | Control | 2.387 (0.97) | 2.989 (1.12) | 146 | 0.392 | 0.952 | 0.028 | |
|  | LessThanHalf | 2.103 (0.49) | 2.286 (1.04) | 14 |  |  |  | |
|  | HalfOrMore | 2.633 (1.52) | 2.962 (1.36) | 60 |  |  |  | |
|  | Complete | 2.429 (0.96) | 3.092 (1.67) | 56 |  |  |  | |

**Supplementary Table 7:**

*Outcome scores and Test Statistics over time based on ITT.*

|  |  | Outcomes scores over time (with imputed values) | | | Effect Size (percentage change) | Test statistics | |
| --- | --- | --- | --- | --- | --- | --- | --- |
| Outcome | Subgroup | Pre-treatment (SD) | Post-treatment (SD) | Follow-up (SD) | PreTx→Post  (∆%, 95%CI) | PreTx→PostTx ∆ p-value | PostTx→Follow-up ∆ p-value |
|  |  |  |  |  |  |  |  |
| CATS_Social | Intervention | 19.328 (8.96) | 21.027 (9.08) | 21.193 (7.68) | -8.8% (-13.1 to -4.5)^1^ | 0.453 | 0.414 |
|  | Control | 19.906 (8.88) | 21.157 (8.4) | 20.976 (8.64) | -6.3% (-13.1 to 0.6) |  |  |
| CATS_Personal_ | Intervention | 17.11 (8.36) | 20.443 (8.86) | 21.446 (7.98) | -19.5% (-24.2 to -14.7) | 0.532 | 0.117 |
|  | Control | 18.135 (9.2) | 21.057 (8.98) | 20.106 (9.53) | -16.1% (-24.1 to -8.1) |  |  |
| SCAS_TOTAL | Intervention | 29.67 (16.02) | 29.376 (16.72) | 29.489 (16.53) | 1% (-4.2 to 6.2) | 0.357 | 0.189 |
|  | Control | 31.753 (16.98) | 31.845 (15.85) | 29.639 (14.79) | -0.3% (-8.4 to 7.8) |  |  |
| UPPS_Urgency | Intervention | 31.219 (6.98) | 30.899 (7.41) | 31.91 (7.58) | 1% (-1.2 to 3.2) | 0.292 | 0.071 |
|  | Control | 27.548 (7.1) | 27.899 (6.71) | 31.536 (6.53) | -1.3% (-5.2 to 2.7) |  |  |
| DES | Intervention | 18.348 (7.32) | 18.679 (6.79) | 19.239 (6.52) | -1.8% (-5.2 to 1.6) | 0.397 | 0.173 |
|  | Control | 18.895 (7.53) | 19.643 (7.44) | 19.325 (6.76) | -4% (-10.3 to 2.4) |  |  |
| SCAS_GAD | Intervention | 6.716 (3.57) | 6.616 (3.66) | 6.441 (3.5) | 1.5% (-3.5 to 6.5) | 0.339 | 0.402 |
|  | Control | 7.342 (3.83) | 6.966 (3.41) | 6.983 (3.41) | 5.1% (-2.4 to 12.7) |  |  |
| SCAS_OCD | Intervention | 5.088 (3.37) | 4.947 (3.37) | 5.054 (3.19) | 2.8% (-3.3 to 8.8) | 0.525 | 0.225 |
|  | Control | 5.253 (3.89) | 4.917 (3.13) | 4.533 (3.11) | 6.4% (-3.3 to 16.1) |  |  |
| SCAS_SA | Intervention | 7.243 (3.6) | 7.227 (3.66) | 6.871 (3.47) | 0.2% (-4.4 to 4.8) | 0.248 | 0.151 |
|  | Control | 7.938 (3.58) | 7.512 (3.54) | 7.517 (3.24) | 5.4% (-1.9 to 12.6) |  |  |
| AFQ | Intervention | 35.768 (12.04) | 36.984 (12.98) | 38.092 (11.86) | -3.4% (-6.7 to -0.1) | 0.156 | 0.173 |
|  | Control | 37.594 (12.66) | 40.933 (12.11) | 39.093 (13.04) | -8.9% (-14.1 to -3.7) |  |  |
| DDI | Intervention | 42.356 (12) | 41.505 (11.34) | 43.394 (11.24) | 2% (-0.4 to 4.5) | 0.427 | 0.179 |
|  | Control | 42.048 (12.23) | 42.17 (11.58) | 40.905 (12.04) | -0.3% (-4.8 to 4.2) |  |  |
| RTQ | Intervention | 23.898 (9.49) | 25.375 (8.94) | 26.983 (9.3) | -6.2% (-9.6 to -2.8) | 0.143 | 0.112 |
|  | Control | 25.238 (10.16) | 28.442 (9.12) | 27.037 (9.28) | -12.7% (-18.6 to -6.8) |  |  |
| BFNE | Intervention | 18.636 (8.21) | 20.331 (7.73) | 21.279 (7.3) | -9.1% (-12.9 to -5.3) | 0.409 | 0.529 |
|  | Control | 19.707 (8.46) | 21.2 (6.85) | 22.471 (8.15) | -7.6% (-13.2 to -1.9) |  |  |
| Pos coping Anger_ | Intervention | 17.872 (4.08) | 17.767 (4.19) | 18.681 (4.1) | 0.6% (-1.6 to 2.7) | 0.424 | 0.189 |
|  | Control | 17.885 (4.1) | 17.864 (4.11) | 18.042 (3.79) | 0.1% (-3.6 to 3.8) |  |  |
| SCSTotal | Intervention | 3.14 (0.58) | 3.018 (0.55) | 3.044 (0.59) | 3.9% (2.3 to 5.5) | 0.506 | 0.316 |
|  | Control | 3.126 (0.62) | 3.041 (0.66) | 3.041 (0.63) | 2.7% (-0.7 to 6.1) |  |  |
| SMFQ_Total | Intervention | 6.128 (5.56) | 8.283 (6.05) | 8.79 (5.46) | -35.2% (-44.2 to -26.1) | 0.24 | 0.389 |
|  | Control | 6.583 (5.7) | 8.4 (5.88) | 8.361 (6.18) | -27.6% (-42.1 to -13.1) |  |  |
| Public Sharing | Intervention | 6.47 (2.48) | 6.495 (2.45) | 5.193 (2.76) | -0.4% (-3.9 to 3.1) | 0.394 | **<0.001** |
|  | Control | 6.649 (1.99) | 6.584 (2.14) | 11.743 (4.82) | 1% (-4.3 to 6.2) |  |  |
| Social Media Surveillance | Intervention | 11.416 (5.13) | 12.202 (5.06) | 13.471 (5.97) | -6.9% (-11 to -2.8) | 0.044 | **<0.001** |
|  | Control | 11.976 (3.98) | 11.693 (4.28) | 18.601 (6.99) | 2.4% (-3.4 to 8.2) |  |  |
| Upset share online | Intervention | 1.637 (1.07) | 1.734 (1.08) | 1.128 (1.06) | -5.9% (-11.9 to 0.2) | **0.006** | **<0.001** |
|  | Control | 1.842 (0.99) | 1.551 (0.81) | 4.654 (2.92) | 15.8% (8.6 to 23) |  |  |
| Text Private | Intervention | 2.168 (1.29) | 2.789 (1.54) | 2.42 (1.51) | -28.7% (-35.2 to -22.2) | 0.38 | **<0.001** |
|  | Control | 2.073 (1.1) | 2.711 (1.1) | 4.835 (2.2) | -30.7% (-39.3 to -22.2) |  |  |
| SM Reduction | Intervention | 2.098 (1.09) | 2.076 (1.11) | 2.241 (1.04) | 1.1% (-3.8 to 5.9) | 0.36 | 0.309 |
|  | Control | 1.941 (1.14) | 2.025 (1.06) | 2.049 (1.16) | -4.3% (-13.2 to 4.5) |  |  |
| FOMO | Intervention | 4.145 (2.03) | 4.589 (2.09) | 4.714 (2.08) | -10.7% (-15.3 to -6.1) | 0.137 | 0.209 |
|  | Control | 4.699 (1.96) | 4.828 (2.2) | 4.86 (2.08) | -2.7% (-10.3 to 4.9) |  |  |
| Neg eff SM | Intervention | 2.666 (2.78) | 3.603 (2.66) | 3.882 (2.47) | -35.1% (-44.3 to -26) | **0.002** | 0.371 |
|  | Control | 3.12 (2.82) | 3.062 (2.62) | 3.325 (2.67) | 1.9% (-11.8 to 15.5) |  |  |
| Mean Actions Online | Intervention | 4.372 (2.16) | 4.999 (2.12) | 5.269 (1.84) | -14.3% (-18.8 to -9.9) | 0.475 | 0.047 |
|  | Control | 4.212 (2.15) | 4.752 (2.3) | 4.328 (1.82) | -12.8% (-21.7 to -4) |  |  |
| SCS SK | Intervention | 5.77 (1.84) | 5.309 (1.75) | 5.17 (1.76) | 8% (5.2 to 10.8) | 0.466 | 0.088 |
|  | Control | 6.164 (1.55) | 5.797 (1.73) | 6.172 (1.65) | 5.9% (1.4 to 10.5) |  |  |
| SCS SJ | Intervention | 6.961 (2.18) | 6.556 (1.96) | 6.67 (1.88) | 5.8% (3.2 to 8.4) | 0.252 | 0.161 |
|  | Control | 6.478 (2.23) | 6.324 (2.29) | 6.106 (2.03) | 2.4% (-3.3 to 8.1) |  |  |
| Common Humanity | Intervention | 5.256 (1.92) | 5.309 (1.74) | 5.492 (1.81) | -1% (-4 to 2) | 0.326 | 0.591 |
|  | Control | 5.829 (1.76) | 5.732 (1.8) | 5.866 (1.61) | 1.7% (-3.4 to 6.7) |  |  |
| Isolation | Intervention | 6.759 (2.08) | 6.401 (1.95) | 6.686 (1.85) | 5.3% (2.7 to 7.9) | 0.23 | 0.133 |
|  | Control | 6.215 (2.08) | 6.136 (2.09) | 5.954 (2.1) | 1.3% (-4.2 to 6.7) |  |  |
| Mindfulness | Intervention | 6.079 (1.92) | 5.773 (1.86) | 5.687 (1.78) | 5% (2.2 to 7.8) | 0.112 | 0.514 |
|  | Control | 6.388 (1.77) | 6.408 (1.81) | 6.433 (1.83) | -0.3% (-4.9 to 4.3) |  |  |
| Overidentification | Intervention | 6.299 (1.53) | 5.987 (1.45) | 6.013 (1.46) | 5% (2.9 to 7.1) | 0.48 | 0.303 |
|  | Control | 6.262 (1.44) | 5.91 (1.62) | 6.086 (1.56) | 5.6% (1.4 to 9.8) |  |  |
| Substance Use | Intervention | 2.371 (1.09) | 2.991 (1.35) | 3.228 (1) | -26.1% (-31.4 to -20.9) | 0.366 | 0.01 |
|  | Control | 2.387 (0.97) | 2.989 (1.12) | 2.789 (1.09) | -25.2% (-32.8 to -17.6) |  |  |

^1^A negative numeric sign implies an increase in scores over time and a positive Δ estimate implies a reduction in scores over time.

| **Supplementary Table 8**  *Sensitivity Analyses for the Effects of Baseline Severity; Considering Time (Pre to Post), Condition and Subgroups Identified by Quantiles* | | | | | | |
| --- | --- | --- | --- | --- | --- | --- |
| Outcome & baseline quantile subgrouping | Cond-ition | Pre-treatment (SD) | Post-treatment (SD) | Time*Condition  (p-value) | Time*baseline severity  (p-value) | Time*Condition*baseline severity  (p-value) |
| IUS Total < 35 | Int* | 23.722 (5.46) | 24.555 (7.09) | ***p<0.001*** | ***p<0.001*** | *p=0.07* |
| IUS Total ≥ 35 (20th↑) | Int | 40 (4.57) | 31.87 (8.63) |  |  |  |
| IUS Total < 35 | Control | 22.735 (5.79) | 26.236 (8.08) |  |  |  |
| IUS Total ≥ 35 (20th↑) | Control | 40.394 (5.48) | 38.375 (9.12) |  |  |  |
| Inhibitory IU < 15 | Int | 9.774 (2.55) | 10.172 (3.17) | ***p<0.001*** | ***p<0.001*** | *p=0.539* |
| Inhibitory IU ≥ 15 (20th↑) | Int | 16.989 (2.07) | 13.136 (3.9) |  |  |  |
| Inhibitory IU < 15 | Control | 8.881 (2.54) | 10.669 (3.6) |  |  |  |
| Inhibitory IU ≥ 15 (20th↑) | Control | 17.486 (2.62) | 15.439 (3.86) |  |  |  |
| Prospective IU < 20 | Int | 13.403 (3.17) | 14.009 (4.28) | ***p<0.001*** | ***p<0.001*** | *p=0.055* |
| Prospective IU ≥ 20 (20th↑) | Int | 22.963 (2.88) | 18.428 (5.25) |  |  |  |
| Prospective IU < 20 | Control | 13.218 (3.41) | 15.07 (4.88) |  |  |  |
| Prospective IU ≥ 20 (20th↑) | Control | 23.139 (3.01) | 21.93 (5.36) |  |  |  |
| CATS Social < 25 | Int | 15.444 (4.24) | 19.954 (8.42) | *p=0.569* | ***p<0.001*** | *p=0.562* |
| CATS Social ≥ 25 (20th↑) | Int | 33.52 (7.18) | 25.062 (10.19) |  |  |  |
| CATS Social < 25 | Control | 15.811 (4.43) | 20.145 (7.48) |  |  |  |
| CATS Social ≥ 25 (20th↑) | Control | 33.235 (6.36) | 24.499 (10.24) |  |  |  |
| CATS Personal < 22 | Int | 13.594 (3.23) | 19.388 (8.43) | *p=0.272* | ***p<0.001*** | *p=0.204* |
| CATS Personal ≥ 22 (20th↑) | Int | 31.562 (7.45) | 24.888 (9.19) |  |  |  |
| CATS Personal < 22 | Control | 13.286 (2.92) | 18.867 (6.82) |  |  |  |
| CATS Personal ≥ 22 (20th↑) | Control | 30.8 (7.9) | 26.744 (11.27) |  |  |  |
| SCAS < 42 | Int | 23.322 (9.38) | 25.994 (14.12) | *p=0.466* | ***p<0.001*** | *p=0.555* |
| SCAS ≥ 42 (20th↑) | Int | 55.198 (10.99) | 42.98 (19.28) |  |  |  |
| SCAS < 42 | Control | 24.321 (9.54) | 28.303 (14.1) |  |  |  |
| SCAS ≥ 42 (20th↑) | Control | 56.235 (12.54) | 43.515 (15.76) |  |  |  |
| Urgency < 36 | Int | 28.113 (5.17) | 29.958 (7.24) | *p=0.345* | ***p<0.001*** | *p=0.435* |
| Urgency ≥ 36 (20th↑) | Int | 39.843 (2.91) | 33.513 (7.26) |  |  |  |
| Urgency < 36 | Control | 25.621 (5.77) | 27.257 (6.54) |  |  |  |
| Urgency ≥ 36 (20th↑) | Control | 38.409 (2.9) | 31.52 (6.47) |  |  |  |
| Envy< 25 | Int | 15.395 (5.07) | 17.827 (6.39) | *p=0.086* | ***p<0.001*** | *p=0.089* |
| Envy ≥ 25 (20th↑) | Int | 28.89 (3.28) | 21.723 (7.3) |  |  |  |
| Envy < 25 | Control | 15.404 (4.87) | 17.883 (6.63) |  |  |  |
| Envy ≥ 25 (20th↑) | Control | 29.278 (3.26) | 24.961 (7.29) |  |  |  |
| GAD SCAS < 10 | Int | 5.385 (2.26) | 5.982 (3.23) | *p=0.323* | ***p<0.001*** | *p=0.391* |
| GAD SCAS ≥ 10 (20th↑) | Int | 12.453 (2.25) | 9.349 (4.11) |  |  |  |
| GAD SCAS < 10 | Control | 5.387 (2.05) | 6.192 (3) |  |  |  |
| GAD SCAS ≥ 10 (20th↑) | Control | 12.525 (2.34) | 9.017 (3.56) |  |  |  |
| OCD SCAS < 8 | Int | 3.762 (1.96) | 4.481 (3.06) | *p=0.531* | ***p<0.001*** | *p=0.519* |
| OCD SCAS ≥ 8 (20th↑) | Int | 10.418 (2.44) | 6.82 (3.87) |  |  |  |
| OCD SCAS < 8 | Control | 3.283 (2.07) | 4.242 (2.75) |  |  |  |
| OCD SCAS ≥ 8 (20th↑) | Control | 10.475 (2.53) | 6.708 (3.37) |  |  |  |
| SA SCAS < 11 | Int | 5.962 (2.53) | 6.772 (3.45) | *p=0.628* | ***p<0.001*** | *p=0.515* |
| SA SCAS ≥ 11 (20th↑) | Int | 12.847 (1.74) | 9.222 (3.86) |  |  |  |
| SA SCAS < 11 | Control | 6.284 (2.3) | 6.874 (3.33) |  |  |  |
| SA SCAS ≥ 11 (20th↑) | Control | 12.811 (1.82) | 9.391 (3.51) |  |  |  |
| AFQ < 46 | Int | 31.093 (7.43) | 35.257 (12.21) | *p=0.046* | ***p<0.001*** | *p=0.569* |
| AFQ ≥ 46 (20th↑) | Int | 54.921 (7.54) | 44.162 (13.65) |  |  |  |
| AFQ < 46 | Control | 31.509 (7.67) | 38.504 (11.1) |  |  |  |
| AFQ ≥ 46 (20th↑) | Control | 54.333 (7.51) | 47.588 (12.33) |  |  |  |
| DDI < 53 | Int | 37.914 (9.05) | 40.532 (11.16) | *p=0.083* | ***p<0.001*** | *p=0.038* |
| DDI ≥ 53 (20th↑) | Int | 58.958 (5.22) | 45.078 (11.35) |  |  |  |
| DDI < 53 | Control | 37.588 (9.18) | 39.846 (10.78) |  |  |  |
| DDI ≥ 53 (20th↑) | Control | 59.267 (5.14) | 50.765 (10.58) |  |  |  |
| RTQ < 33 | Int | 20.099 (6.67) | 24.499 (8.53) | *p=0.113* | ***p<0.001*** | *p=0.404* |
| RTQ ≥ 33 (20th↑) | Int | 37.622 (4.1) | 28.683 (9.68) |  |  |  |
| RTQ < 33 | Control | 20.741 (7.05) | 27.029 (8.89) |  |  |  |
| RTQ ≥ 33 (20th↑) | Control | 38.8 (5.06) | 32.583 (8.67) |  |  |  |
| BFNE < 26 | Int | 15.391 (5.33) | 19.33 (7.5) | *p=0.443* | ***p<0.001*** | *p=0.61* |
| BFNE ≥ 26 (20th↑) | Int | 31.28 (4.36) | 24.339 (7.28) |  |  |  |
| BFNE < 26 | Control | 15.654 (5.31) | 19.855 (6.34) |  |  |  |
| BFNE ≥ 26 (20th↑) | Control | 31.378 (3.81) | 24.888 (7.01) |  |  |  |
| Pos coping Anger < 21 | Int | 16.243 (2.95) | 17.276 (4.06) | *p=0.309* | ***p<0.001*** | *p=0.147* |
| Pos coping Anger ≥ 21 (20th↑) | Int | 23.245 (2.34) | 19.32 (4.19) |  |  |  |
| Pos coping Anger < 21 | Control | 16.103 (2.96) | 16.974 (3.64) |  |  |  |
| Pos coping Anger ≥ 21 (20th↑) | Control | 23.054 (2.03) | 20.355 (4.4) |  |  |  |
| SCS < 4 | Int | 2.965 (0.46) | 2.992 (0.54) | *p=0.04* | ***p<0.001*** | ***p=0.013*** |
| SCS ≥ 4 (20th↑) | Int | 4.014 (0.28) | 3.122 (0.57) |  |  |  |
| SCS < 4 | Control | 2.967 (0.49) | 2.949 (0.63) |  |  |  |
| SCS ≥ 4 (20th↑) | Control | 4.117 (0.32) | 3.636 (0.55) |  |  |  |
| SMFQ < 11 | Int | 3.88 (2.97) | 7.54 (5.69) | *p=0.269* | ***p<0.001*** | *p=0.441* |
| SMFQ ≥ 11 (20th↑) | Int | 15.471 (3.96) | 11.579 (6.44) |  |  |  |
| SMFQ < 11 | Control | 3.918 (3.03) | 7.454 (5.38) |  |  |  |
| SMFQ ≥ 11 (20th↑) | Control | 15.029 (3.72) | 11.512 (6.4) |  |  |  |
| Public Sharing < 8 | Int | 5.083 (1.53) | 6.156 (2.45) | *p=0.448* | ***p<0.001*** | *p=0.024* |
| Public Sharing ≥ 8 (20th↑) | Int | 9.058 (1.77) | 7.177 (2.34) |  |  |  |
| Public Sharing < 8 | Control | 5.505 (1.33) | 6.085 (2.08) |  |  |  |
| Public Sharing ≥ 8 (20th↑) | Control | 8.75 (1) | 7.49 (1.94) |  |  |  |
| Surveillance SM < 15 | Int | 9.178 (3.47) | 11.707 (4.91) | *p=0.474* | ***p<0.001*** | ***p=0.001*** |
| Surveillance SM ≥ 15 (20th↑) | Int | 17.729 (3.64) | 13.716 (5.22) |  |  |  |
| Surveillance SM < 15 | Control | 10.327 (3.01) | 10.833 (4.12) |  |  |  |
| Surveillance SM ≥ 15 (20th↑) | Control | 16.73 (2.24) | 14.168 (3.79) |  |  |  |
| Upset share online < 3 | Int | 1.22 (0.41) | 1.732 (1.09) | *p=0.652* | ***p<0.001*** | *p=0.022* |
| Upset share online ≥ 3 (20th↑) | Int | 3.556 (1.08) | 1.749 (1.06) |  |  |  |
| Upset share online < 3 | Control | 1.33 (0.47) | 1.469 (0.77) |  |  |  |
| Upset share online ≥ 3 (20th↑) | Control | 3.256 (0.54) | 1.759 (0.89) |  |  |  |
| Textpriv < 3 | Int | 1.435 (0.5) | 2.701 (1.52) | *p=0.414* | ***p<0.001*** | *p=0.377* |
| Textpriv ≥ 3 (20th↑) | Int | 3.623 (1.13) | 2.965 (1.57) |  |  |  |
| Textpriv < 3 | Control | 1.385 (0.49) | 2.609 (1.08) |  |  |  |
| Textpriv ≥ 3 (20th↑) | Control | 3.417 (0.61) | 2.91 (1.12) |  |  |  |
| SM Reduction< 3 | Int | 1.339 (0.47) | 1.964 (1.09) | *p=0.618* | ***p<0.001*** | *p=0.706* |
| SM Reduction≥ 3 (20th↑) | Int | 3.299 (0.58) | 2.254 (1.11) |  |  |  |
| SM Reduction< 3 | Control | 1.26 (0.44) | 1.854 (0.96) |  |  |  |
| SM Reduction ≥ 3 (20th↑) | Control | 3.444 (0.72) | 2.39 (1.17) |  |  |  |
| FOMO < 6 | Int | 3.195 (1.14) | 4.348 (2.05) | *p=0.489* | ***p<0.001*** | *p=0.025* |
| FOMO ≥ 6 (20th↑) | Int | 7.109 (1.16) | 5.432 (1.98) |  |  |  |
| FOMO < 6 | Control | 3.552 (1.13) | 4.228 (1.97) |  |  |  |
| FOMO ≥ 6 (20th↑) | Control | 6.959 (1.13) | 6.013 (2.17) |  |  |  |
| Neg eff SM < 5 | Int | 1.422 (1.81) | 3.264 (2.54) | *p=0.018* | ***p<0.001*** | *p=0.172* |
| Neg eff SM ≥ 5 (20th↑) | Int | 6.411 (1.59) | 4.749 (2.64) |  |  |  |
| Neg eff SM < 5 | Control | 1.819 (1.9) | 2.483 (2.37) |  |  |  |
| Neg eff SM ≥ 5 (20th↑) | Control | 6.55 (1.79) | 4.494 (2.67) |  |  |  |
| Mean acts online < 6 | Int | 3.326 (0.63) | 4.777 (2) | *p=0.563* | ***p<0.001*** | *p=0.291* |
| Mean acts online ≥ 6 (20th↑) | Int | 7.923 (1.71) | 5.75 (2.34) |  |  |  |
| Mean acts online < 6 | Control | 3.322 (0.61) | 4.437 (2.09) |  |  |  |
| Mean acts online ≥ 6 (20th↑) | Control | 8.074 (2.22) | 6.115 (2.66) |  |  |  |
| SCS SK < 7 | Int | 4.785 (1.32) | 5.162 (1.75) | *p=0.043* | ***p<0.001*** | ***p=0.006*** |
| SCS SK ≥ 7 (20th↑) | Int | 7.776 (0.91) | 5.608 (1.72) |  |  |  |
| SCS SK < 7 | Control | 5.143 (1.04) | 5.443 (1.72) |  |  |  |
| SCS SK ≥ 7 (20th↑) | Control | 7.632 (0.74) | 6.349 (1.65) |  |  |  |
| SCS SJ < 9 | Int | 5.929 (1.64) | 6.311 (1.91) | *p=0.264* | ***p<0.001*** | *p=0.113* |
| SCS SJ ≥ 9 (20th↑) | Int | 9.659 (0.47) | 7.127 (1.96) |  |  |  |
| SCS SJ < 9 | Control | 5.75 (1.76) | 5.982 (2.17) |  |  |  |
| SCS SJ ≥ 9 (20th↑) | Control | 9.88 (0.33) | 8.017 (2.05) |  |  |  |
| Common Humanity < 7 | Int | 4.41 (1.44) | 5.209 (1.72) | *p=0.373* | ***p<0.001*** | *p=0.109* |
| Common Humanity ≥ 7 (20th↑) | Int | 7.619 (0.8) | 5.515 (1.76) |  |  |  |
| Common Humanity < 7 | Control | 4.894 (1.18) | 5.524 (1.72) |  |  |  |
| Common Humanity ≥ 7 (20th↑) | Control | 7.723 (1.01) | 6.143 (1.9) |  |  |  |
| SCS Iso < 8 | Int | 5.442 (1.43) | 6.1 (1.89) | *p=0.297* | ***p<0.001*** | *p=0.204* |
| SCS Iso ≥ 8 (20th↑) | Int | 8.893 (0.88) | 6.883 (1.96) |  |  |  |
| SCS Iso < 8 | Control | 5.15 (1.34) | 5.658 (2) |  |  |  |
| SCS Iso ≥ 8 (20th↑) | Control | 8.854 (0.9) | 7.235 (1.91) |  |  |  |
| SCS Mind < 8 | Int | 5.297 (1.54) | 5.636 (1.88) | *p=0.022* | ***p<0.001*** | *p=0.334* |
| SCS Mind ≥ 8 (20th↑) | Int | 8.393 (0.69) | 6.159 (1.74) |  |  |  |
| SCS Mind < 8 | Control | 5.53 (1.27) | 6.189 (1.72) |  |  |  |
| SCS Mind ≥ 8 (20th↑) | Control | 8.488 (0.77) | 6.955 (1.93) |  |  |  |
| SCS OI < 8 | Int | 5.732 (1.12) | 5.94 (1.41) | *p=0.272* | ***p<0.001*** | *p=0.029* |
| SCS OI ≥ 8 (20th↑) | Int | 8.495 (0.67) | 6.128 (1.57) |  |  |  |
| SCS OI < 8 | Control | 5.735 (1) | 5.706 (1.47) |  |  |  |
| SCS OI ≥ 8 (20th↑) | Control | 8.393 (0.73) | 6.847 (1.83) |  |  |  |
| Substance < 2 | Int | 2.342 (1.09) | 2.984 (1.36) | *p=0.459* | ***p<0.001*** | *p<0.001* |
| Substance ≥ 2 (20th↑) | Control | 2.341 (0.95) | 3.002 (1.13) |  |  |  |

*Int Intervention

**Supplementary Table 9**

*Social Media Items*

|  |  | **Mean** | **Variance** |
| --- | --- | --- | --- |
| Public Sharing | When I have a good time it is important for me to share the details online | 2.33 | 1.09 |
|  | write a status update | 2.11 | 1.83 |
|  | post photos | 2.96 | 1.49 |
| Surveillance SM | On social media how often do you read the newsfeed | 4.39 | 1.04 |
|  | On social media how often do you read a friend’s status update | 7.15 | 2.76 |
|  | On social media how often do you view a friend’s photo | 5.43 | 2.25 |
|  | On social media how often do you browse a friend’s timeline | 8.22 | 2.01 |
| Upset Share | On social media how often do you go online to share things that have upset you? | 1.78 | 2.27 |
| Text private | On social media how often do you Text friends privately to share things that have upset you? | 4.4 | 2.34 |
| Social Media Reduction | I use social media less now because it often made me feel inadequate | 4.07 | 1.18 |
| FOMO | I am afraid that I will miss out on something if I don’t stay connected to my online social networks. | 2.45 | 1.45 |
|  | I feel worried and uncomfortable when I can’t access my social media accounts. | 2.02 | 1.28 |
| Neg Eff of SM | I find it difficult to relax or sleep after spending time on social networking sites. | 1.85 | 1.09 |
|  | I feel my brain ‘burnout’ with the constant connectivity of social media. | 1.75 | 0.98 |
|  | I notice I feel envy when I use social media. | 2.27 | 1.63 |
|  | I can easily detach from the envy that appears following the use of social media (reverse scored) | 2.9 | 1.87 |
| Mean acts online | Feeling envious about another person has led me to post a comment online about another person to make them laugh | 1.45 | 0.77 |
|  | Feeling envious has led me to post a photo online without someone’s permission to make them angry or to make fun of them | 1.32 | 0.58 |
|  | Feeling envious has prompted me to keep another student out of things on purpose, excluding her from my group of friends or ignoring her | 1.55 | 0.86 |

**Supplementary Table References**

Angold, A., Costello, E. J., Messer, S. C., & Pickles, A. (1995). Development of a short questionnaire for use in epidemiological studies of depression in children and adolescents. *International Journal of Methods in Psychiatric Research, 5(4),* 237–249.

Australian Psychological Society. (2015). *Stress and Wellbeing In Australia Survey.* Melbourne, Australia. https://www.headsup.org.au/docs/default-source/default-document-library/stress-and-wellbeing-in-australia-report.pdf?sfvrsn=7f08274d_4

Furlong, M. J., Smith, D. C., & Bates, M. P. (2002). Further development of the Multidimensional School Anger Inventory: Construct validation, extension to female adolescents, and preliminary norms. Journal of Psychoeducational Assessment, 20(1), 46-65. <https://doi.org/10.1177/073428290202000104>

Greco, L. A., Lambert, W., & Baer, R. A. (2008). Psychological Inflexibility in Childhood and Adolescence: Development and Evaluation of the Avoidance and Fusion Questionnaire for Youth. *Psychological Assessment*, *20*(2), 93–102. <https://doi.org/10.1037/1040-3590.20.2.9>

Kahn, J. H., & Hessling, R. M. (2001). Measuring the tendency to conceal versus disclose psychological distress. *Journal of Social & Clinical Psychology*, 20(1), 41–65. <https://doi.org/10.1521/jscp.20.1.41.22254>

McBride, N., Farringdon, F. & Midford, R. (2000) What harms do young Australians experience in alcohol use situations. Australian & New Zealand Journal of Public Health, 24, 54–60 <https://doi.org/10.1111/j.1467-842x.2000.tb00723.x>

McEvoy, P. M., Salmon, K., Hyett, M. P., Jose, P. E., Gutenbrunner, C., Bryson, K., & Dewhirst, M. (2019). Repetitive negative thinking as a transdiagnostic predictor of depression and anxiety symptoms in adolescents. Assessment, 26(2), 324-335. <https://doi.org/10.1177/1073191117693923>

McEvoy, P.M., Thibodeau, M.A., Asmundson, G.J.G. (2014) Trait Repetitive Negative Thinking: A brief transdiagnostic assessment. Journal of Experimental Psychopathology, 5, 1-17. Doi. [10.5127/jep.037813](doi:10.5127/jep.037813)

Przybylski, A. K., Murayama, K., DeHaan, C. R., & Gladwell, V. (2013). Motivational, emotional, and behavioral correlates of fear of missing out. Computers in Human Behavior, 29, 1841-1848. <https://doi.org/10.1016/j.chb.2013.02.014>

Raes, F., Pommier, E., Neff, K. D., & Van Gucht, D. (2011). Construction and factorial validation of a short form of the self‐compassion scale. *Clinical Psychology & Psychotherapy*, *18*(3), 250-255. <https://doi.org/10.1002/cpp.702>

Rodebaugh, T. L., Woods, C. M., Thissen, D. M., Heimberg, R. G., Chambless, D. L., & Rapee, R. M. (2004). More information from fewer questions: the factor structure and item properties of the original and brief fear of negative evaluation scale. Psychological assessment, 16(2), 169. <https://doi.org/10.1037/10403590.16.2.169>

Schniering, C. A., & Rapee, R. M. (2002). Development and validation of a measure of children’s automatic thoughts: the children’s automatic thoughts scale. *Behaviour Research & Therapy*, 40(9), 1091-1109. <https://doi.org/10.1016/S0005-7967(02)00022-0>

Smith, R. H., Parrott, W. G., Diener, E. F., Hoyle, R. H., & Kim, S. H. (1999). Dispositional envy. *Personality & Social Psychology Bulletin*, *25*(8), 1007-1020. <https://doi.org/10.1177/01461672992511008>

Spence, S. H. (1998). A measure of anxiety symptoms among children. *Behaviour Research and Therapy*, 36(5), 545-566. [https://doi.org/10.1016/S0005-7967(98)00034-5](about:blank)

Tandoc, E. C., Ferrucci, P., & Duffy, M. (2015). Facebook use, envy, and depression among college students: Is facebooking depressing? *Computers in Human Behavior,* 43, 139–146. <https://doi.org/10.1016/j.chb.2014.10.053>

Whiteside, S. P., & Lynam, D. R. (2001). The Five Factor Model and impulsivity: using a structural model of personality to understand impulsivity. *Personality and Individual Differences*, *30*(4), 669–689. <https://doi.org/10.1016/S0191-8869(00)00064-7>
